# Regional venous-to-arterial carbon dioxide pressure and content differences during endotoxemic shock: influence of hydrogen ion accumulation vs. Haldane effect

**DOI:** 10.1186/s40635-025-00805-0

**Published:** 2025-09-08

**Authors:** Gustavo A. Ospina-Tascón, Daniel De Backer, José L. Aldana, Alberto F. García Marín, Luis E. Calderón, Julián Chica, Gustavo García-Gallardo, Nicolás Orozco, Jihad Mallat

**Affiliations:** 1 Department of Intensive Care, Fundación Valle del Lili, Cali, Colombia; 2 Translational Research Laboratory in Critical Care Medicine (TransLab-CCM), Universidad Icesi, Cali, Colombia; 3 Intensive Care Department. CHIREC Hospitals, Université Libre de Bruxelles, Brussels, Belgium; 4 Critical Care Division, Integrated Hospital Care Institute, Cleveland Clinic Abu Dhabi, Abu Dhabi, United Arab Emirates; 5 Cleveland Clinic Lerner College of Medicine of Case Western Reserve University, Cleveland, OH USA; 6 Faculty of Medicine, University of Banja Luka, Banja Luka, Republic of Srpska Bosnia and Herzegovina

**Keywords:** CO_2_ content, Douglas equation, Haldane effect, PCO_2_ gaps, PCO_2_:CCO_2_ dissociation curve

## Abstract

**Background:**

The relationship between carbon dioxide pressures (PCO_2_) and contents (CCO_2_) is linked to the Haldane effect. Nevertheless, under shock conditions, hydrogen ion accumulation might strongly influence the discrepancies between PCO_2_ and CCO_2_. This study aims to evaluate the impact of hydrogen ion accumulation and hemoglobin oxygen saturation (Haldane effect) on PCO_2_:CCO_2_ relationships during induction and resuscitation of endotoxemic shock.

**Methods:**

Shock was induced by an escalating dose of lipopolysaccharide in 12 female Landrace pigs. Norepinephrine was then started to maintain mean arterial pressure ≥ 75 mmHg, while successive fluid boluses were administered targeting arterial lactate < 2.0 mmol·L^−1^ or decreases > 10% per 30 min. Mesenteric venous and arterial PCO_2_ were measured at baseline, time of shock, and then, every hour for 6 h, while their respective CCO_2_ were computed using the Douglas equation. Mesenteric venous-to-arterial PCO_2_ and CCO_2_ differences (i.e., ΔPCO_2_ and ΔCCO_2_), and then, their absolute arithmetic differences (i.e., [|ΔPCO_2_ – ΔCCO_2_|]) were calculated. Discrepancies in [|ΔPCO_2_ – ΔCCO_2_|] between adjacent measurement time points (i.e., ∆–[|ΔPCO_2_ – ΔCCO_2_|]) were compared with the variations in mesenteric venous O_2_ saturation (∆–S_vmes_O_2_) and arterial-to-mesenteric venous pH (∆–pH_a-vmes_). In addition, arterial and venous CCO_2_ values were recalculated, maintaining baseline pH (Def_pH_) or SO_2_ values (Def_SO2_) to then quantify the impact of pH and S_vmes_O_2_ on the PCO_2_:CCO_2_ relationship.

**Results:**

Variations in ∆–[|∆PCO_2_ – ∆CCO_2_|]) were paralleled by ∆–pH_a-vmes_ (R^2^ = 0.56, p < 0.001), while poorly correlated with ∆–S_vmes_O_2_ (R^2^ = 0.15, p < 0.001). When variations in pH were not included in CCO_2_ calculations (i.e., Def_pH_–CCO_2_), both arterial and mesenteric venous CCO_2_ disagreed in ranges from 21.8 to 50.4% and 15.3 to 47.6%, respectively. Conversely, overestimation of CCO_2_ was almost null when variations in SvmesO_2_ were not assumed (Def_SvmesO2_). Calculations under Def_pH_–CCO_2_ conditions revealed an almost linear relationship between PCO_2_ and CCO_2_, contrasting with a non-linear relationship when pH variations were acknowledged.

**Conclusions:**

Regional splanchnic PCO_2_:CCO_2_ relationship was mostly influenced by hydrogen ion accumulation rather than the Haldane effect during development and resuscitation of endotoxemic shock. Predominant influence of hydrogen ion accumulation on PCO_2_:CCO_2_ dissociation curve during endotoxemic shock could have important implications when interpreting ΔPCO_2_ and its combination with arterial-to-venous oxygen differences in vasodilated shock conditions.

**Supplementary Information:**

The online version contains supplementary material available at 10.1186/s40635-025-00805-0.

## Background

According to the Fick equation, there is an inverse hyperbolic relationship between cardiac output and the difference between venous-to-arterial carbon dioxide (CO_2_) content (ΔCCO_2_) for a given total CO_2_ production. Consequently, a low cardiac output results in a reduced peripheral washout of the CO_2_ generated by the tissues (CO_2_ stagnation), which in turn causes increases in ΔCCO_2_. Nevertheless, the computation of CO_2_ content (CCO_2_) is cumbersome and subject to a high risk of errors. Despite a global curvilinear shape of the relation between CO_2_ partial pressure (PCO_2_) and the total CCO_2_ (i.e., the PCO_2_:CCO_2_ relationship), there is a relatively linear association between CCO_2_ and PCO_2_ over normal physiological ranges of CO_2_ contents so that PCO_2_ is usually considered as a surrogate of CCO_2_ [1–4].

Both experimental and clinical studies have emphasized on the close relationship between venous-to-arterial PCO_2_ difference (ΔPCO_2_) and cardiac output [5–14], and also between ΔPCO_2_ and microcirculatory blood flow distribution [15]. Thus, ΔPCO_2_ has been considered a marker of adequacy of flow to the tissues [16–18], having been used to assess tissue hypoperfusion in both septic shock [19–24] and high-risk surgical patients [25]. Moreover, the combination of ΔPCO_2_ or ΔCCO_2_ with arterial-to-venous oxygen differences (i.e., the ΔPCO_2_:Ca-vO_2_ and ΔCCO_2_:Ca-vO_2_ ratios) might provide additional information to lactate measurements in septic shock [26–31] and could predict increases in oxygen consumption in fluid responders [32]. Nevertheless, the clinical utility of ΔPCO_2_:Ca-vO_2_ and ΔCCO_2_:Ca-vO_2_ ratios might be hindered by their intrinsic physiological complexities, which include, among others, the potential influence of the Haldane effect on PCO_2_:CCO_2_ relationships, especially under dynamic conditions as occurred during resuscitation of shock states. Indeed, clinical observations reveal some opposite results when interpreting the association of ΔPCO_2_:Ca-vO_2_ and ΔCCO_2_:Ca-vO_2_ ratios with clinical outcomes [26–31].

Data about factors influencing the PCO_2_:CCO_2_ relationship are conflicting, with studies showing a significant influence of metabolic acidosis [33], while others suggest a significant influence of the Haldane effect [34]. Accordingly, evaluating the extent to which these factors impact the PCO_2_:CCO_2_ dissociation curve might provide a better understanding of the interpretation of ΔPCO_2_ and ΔCCO_2_ and their applicability during resuscitation of shock states. In this post-hoc analysis of an experimental endotoxemic model [35], we aimed to investigate the impact of hydrogen ion accumulation vs. venous oxygen saturation (Haldane effect) on the PCO_2_:CCO_2_ relationship during the induction and early resuscitation phases of vasodilated shock.

## Methods

### Animal preparation and anesthesia

The present study was approved by the Institutional Animal Research Committee (CIECUAE 0021/2019; Universidad Icesi, Cali, Colombia) [35]. All the experiments were conducted in accordance with the ARRIVE guidelines (see Supplemental Material) and the International Guiding Principles for Biomedical Research Involving Animals [36], which align with national regulations regarding the care of animals used for experimental purposes. Twelve female Landrace pigs (32–38 kg) underwent endotoxemic shock by lipopolysaccharide infusion. All procedures regarding animal care, anesthesia, ventilatory support, and general monitoring are described elsewhere (see supplemental material) [35].

### Surgical preparation/monitoring installation

Neck vessels were accessed by surgical dissection; catheters were then inserted in the ascending aorta through the carotid artery (Bi-lumen central venous 7-Fr catheter; CV-17702. Arrow International, Reading, PA. USA) and left internal jugular vein (Three-lumen central venous 7-Fr catheter; CV-25703. Arrow International Reading, PA. USA), and a continuous-cardiac-output pulmonary artery catheter (7.5-Fr, Edwards Swan-Ganz CCO; Baxter Edwards Critical Care. Irvine, CA. USA) was inserted through the right internal jugular vein to monitor pulmonary arterial pressure, pulmonary artery occlusion pressure, and to withdraw mixed-venous blood samples. A thermistor-tipped catheter was inserted through the right femoral artery and connected to a transpulmonary–thermodilution system (PulsioFlex-PiCCO; PULSION Medical Systems AG; Munich, Germany). Continuous electrocardiogram, pulse oximetry, and invasive arterial pressures were also monitored throughout the experiment (Drägger Infinity Vista XL; Drägger Medical System, Lübeck, Germany) as described elsewhere [35] (also see supplemental material).

Ultrasound Doppler flow probes (Transonic Systems Inc., Ithaca NY., USA) were placed around the supra-celiac abdominal aorta and superior mesenteric artery and connected to ultrasound flowmeter modules (Transonic perivascular flow module TS420; Transonic Systems Inc., Ithaca NY., USA), while small laser Doppler (LDF) probes were attached to the serosa and mucosa surfaces of the proximal jejunum, 30 to 40 cm from the ligament of Treitz (OxyFlo Pro. Oxford Optronix). All flow signals were continuously recorded on a laptop (HP ProBook 440 G4. Hewlett Packard Development Company, LP; Palo Alto, CA. USA) using a data acquisition system (PowerLab 4/35; Ad Instruments). In addition, a double-lumen catheter (2-lm central venous 4-Fr bi-lumen catheter; CS-14402. Arrow International. Morrisville, NC. USA) was inserted through the splenic vein up to the confluence with the superior mesenteric vein. Then, splenectomy was performed after arterial local constriction with epinephrine. Finally, microvascular blood flow at the jejunal mucosa was assessed by portable video microscopy (see supplemental material).

### General monitoring

Arterial pressures were simultaneously recorded at the ascending aorta and femoral arteries (Drägger Infinity Vista XL; Drägger Medical System, Lübeck, Germany), while cardiac output was measured by transpulmonary thermodilution (PulsioFlex-PiCCO; PULSION Medical Systems AG; Munich, Germany) and calibrated with the femoral pulse-contour to subsequently estimate stroke volume and pulse pressure variations. Calibrations were performed every hour during the entire experiment by 10 mL boluses of normal saline 0.9% solution at 4–6 ºC injected through the jugular venous catheter. Pulmonary artery and pulmonary arterial occlusion pressures were closely monitored during endotoxin infusion and the rest of the experiment (for more details, see supplemental material).

### Experimental protocol

The timeline of the experiment is depicted in Figure S1. A 60-min stabilization was ensured after surgical preparation, catheter placement, and monitoring installation. Baseline measurements (BL) were then performed and endotoxic shock was induced by intravenous infusion of lipopolysaccharide (*Escherichia coli* O55:B5 purified by gel-filtration chromatography; Sigma-Aldrich; Saint Louis, MO. USA), starting at 0.5 µgr·kg-1·min^−1^ and escalating progressively until 6 µgr·kg^−1^·min^−1^ over around 4 h (for more details, see supplemental material) [35]. Pulmonary pressure was carefully monitored during lipopolysaccharide dose escalation to avoid severe pulmonary hypertension and right ventricular failure. Shock (TS) was declared when mean arterial pressure (MAP) was steadily under 60 mmHg for at least 30 min, and arterial and mesenteric lactate concentrations were ≥ 2.0 mmol/L. Lipopolysaccharide infusion was maintained up to 30 min after TS. Resuscitation was then started with fluids and norepinephrine as described elsewhere [35]. Once MAP ≥ 75 mmHg was achieved, successive mini-fluid boluses of 4 mL·kg^−1^ of Lactate Ringer were administered aiming to increase cardiac preload when positive fluid responsiveness was predicted (i.e., when pulse pressure [PPV] and stroke volume variations [SVV] were ≥ 15%), targeting arterial lactate levels < 2.0 mmol·L^−1^ and/or lactate decrease of at least 10% per 30 min. All hemodynamics, respiratory parameters, and both systemic and regional blood gas analyses were performed at baseline, TS, when achieving MAP ≥ 75 mmHg, and then, every hour during the next 6 h. Finally, euthanasia was performed at the end of the experiment according to the local regulations for animal research (see supplemental material).

### ***Calculations of CO***_***2***_***-derived variables***

Simultaneous arterial and mesenteric venous blood samples were withdrawn to measure blood gases, hemoglobin, and lactate concentrations (GEM 5000 Premier, Instrumentation Laboratory. Bedford, MA. USA.). CO_2_-derived parameters were calculated according to the following equations:

1 $${\text{Mesenteric}} - {\text{venous}} - {\text{to}} - {\text{arterial PCO}}_{{2}} {\text{difference }}(\Delta {\text{PCO}}_{{2}} )\, = \,{\text{PvmesCO}}_{{2}} {-}{\text{PaCO}}_{{2}}$$

where PvmesCO_2_ and PaCO_2_ represent the mesenteric venous PCO_2_ and arterial PCO_2_, respectively. Mesenteric venous-to-arterial CO_2_ content differences (∆CCO_2_) were estimated according to Douglas et al. [31], which includes pH and oxygen saturation:

2 $${\text{Blood CO}}_{{2}} {\text{content }}\left[ {{\text{blood Douglas CCO}}_{{2}} \left( {{\text{ml}}} \right)} \right]\, = \,{\text{Plasma CCO}}_{{2}} \, \times \,\left[ {{1}{-}0.0{289}\, \times \,\left( {{\text{Hb}}} \right)/\left( {{3}.{352}{-}0.{456}\, \times \,{\text{SO}}_{{2}} } \right)\, \times \,\left( {{8}.{142}{-}{\text{pH}}} \right)} \right]$$

3 $${\text{where }}\,{\text{plasma}}\,CCO_{2} \, = \,2.226 \times S \times plasma\,PCO_{2} \, \times (1 + 10^{PH - PK^{\prime}} )$$

Here, CCO_2_ represents CO_2_ content, SO_2_ is the oxygen saturation, S is the plasma CO_2_ solubility coefficient, and pK’ is the apparent pK.

Meanwhile, S and pK’ were calculated as follows:

4 $${\text{S}}\, = \,0.0{3}0{7}\, + \,\left[ {0.000{57}\, \times \,\left( {{37}{-}T} \right)} \right]\, + \,\left[ {0.0000{2}\, \times \,\left( {{37}{-}T} \right)^{2} } \right]$$

5 $${\text{and}}\,{\text{pK}}{\prime} \,{ = 6}{\text{.086}}\,{ + }\left[ {0.042 \times \left( {7.4 - {\text{pH}}} \right)} \right] + \left( {\left( {38 - {\text{T}}} \right) \times \left\{ {0.00472 + \left[ {0.00139 \times \left( {7.4 - {\text{pH}}} \right)} \right]} \right\}} \right)$$

where T is the temperature expressed as °C.

Thus, the difference between mesenteric venous-to-arterial CCO_2_ was determined as

6 $$\Delta {\text{CCO}}_{{2}} \, = \,{\text{CvmesCO2}}\, - \,{\text{CaCO}}_{{2}}$$

where CvmesCO_2_ is the mesenteric venous CO_2_ content, and CaCO_2_ is the arterial CO_2_ content.

### ***Regional ΔPCO***_***2***_***:Ca-vO***_***2***_*** and ΔCCO***_***2***_***:Ca-vO***_***2***_*** ratios***

Regional splanchnic respiratory quotients were calculated as the combination of ΔPCO_2_ and ΔCCO_2_ with their arterial-to-mesenteric venous oxygen content differences:

7 $${\text{Ca}} - {\text{vO}}_{{2}} \, = \,[({\text{Hb}}\, \times \,{1}.{34}\, \times \,{\text{S}}_{{\text{a}}} {\text{O}}_{{2}} )\, + \,\left( {{\text{P}}_{{\text{a}}} {\text{O}}_{{2}} \, \times \,0.00{3}} \right)\left] {\, - \,[({\text{Hb}}\, \times \,{1}.{34}\, \times \,{\text{S}}_{{{\text{vmes}}}} {\text{O}}_{{2}} )\, + \,\left( {{\text{P}}_{{{\text{vmes}}}} {\text{O}}_{{2}} \, \times \,0.00{3}} \right)} \right]$$

where Hb denotes hemoglobin concentration; S_a_O_2_, arterial oxygen saturation; P_a_O_2_, arterial oxygen partial pressure; S_vmes_O_2_, mesenteric venous oxygen saturation; and P_vmes_O_2_, mesenteric venous oxygen partial pressure.

### The influence of hydrogen ion accumulation versus the Haldane effect

Two approaches were used to determine the influence of the Haldane effect vs. hydrogen ion accumulation on the dynamic variations of the PCO_2_:CCO_2_ relationship: first, ΔCCO_2_ to ΔPCO_2_ discrepancies were calculated between pairs of adjacent time-point measurements to plot them against variations in SmesO_2_ and variations in arterial-to-venous pH; second, the time course of arterial and venous CCO_2_ values were recalculated maintaining baseline pH and SvO_2_, i.e., default pH and default SO_2_ values.

### ***ΔCCO***_***2***_*** to ΔPCO***_***2***_*** discrepancies (∆–[|ΔPCO***_***2***_*** – ΔCCO***_***2***_***|])***

Absolute arithmetic differences between ΔPCO_2_ and ΔCCO_2_ (i.e., [|ΔPCO_2_ – ΔCCO_2_|]) and arterial-to-mesenteric venous pH differences (Δ–pH_a-vmes_) were calculated at every time point of measurements from baseline until the end of the resuscitative period. Then, variations of [|ΔPCO_2_ – ΔCCO_2_|] between two adjacent time-point measurements (from baseline till shock, from shock till T1, T1 till T2, and so on), i.e., the ∆–[|ΔPCO_2_ – ΔCCO_2_|], were plotted against their respective variations in arterial-to-mesenteric venous pH differences (Δ–pH_a-vmes_) and mesenteric venous oxygen saturation (∆–S_vmes_O_2_), aiming to evaluate the effect of hydrogen ion accumulation vs. the impact of SmesO_2_ (Haldane effect) on the PCO_2_:CCO_2_ relationship.

### ***Default values of CaCO***_***2***_***, CvCO***_***2,***_*** and ΔCCO***_***2***_

Default (Def) values of CCO_2_ were calculated with Douglas’s equation [37] to compare the magnitude of CCO_2_ discrepancies caused by not acknowledging changes in pH or oxygen saturation (SO_2_) (i.e., metabolic acidosis vs. Haldane effect) on the PCO_2_:CCO_2_ relationship from baseline to the different time-point measurements. Such default CCO_2_ values represent calculations performed using only the baseline values of pH and SO_2_. Thus, consequently,

8 $${\text{Def}}_{{{\text{pH}}}} \Delta {\text{CCO}}_{{2}} \, = \,{\text{Def}}_{{{\text{pHvmes}}}} {\text{CvmesCO}}_{{2}} \, - \,{\text{Def}}_{{{\text{pH}}}} {\text{CaCO}}_{{2}}$$

9 $${\text{ and Def}}_{{{\text{SO2}}}} \Delta {\text{CCO}}_{{2}} \, = \,{\text{Def}}_{{{\text{SO2}}}} {\text{CvmesCO}}_{{2}} \, - \,{\text{Def}}_{{{\text{SO2}}}} {\text{CaCO}}_{{2}}$$

Def values are so named because they do not account for changes in one or more of the independent variables at every measurement time point. For example, if the changing PCO_2_ and SO_2_ values were used to calculate CCO_2_ at every time point, but the pH remained at its baseline (or Def) value, the CCO_2_ is identified as Def_pH_CCO_2_. Similarly, we calculated Def_SO2_ when changes in SO_2_ values were not considered. The percentual discrepancies of arterial and mesenteric venous Def_pH_ and Def_SO2_ values from actual CaCO_2_ and CvmesCO_2_ were calculated at every measurement time point as

10 $$\% {\text{ discrepancy}}\, = \,{1}00\, \times \,\left( {{\text{default}}\, - \,{\text{baseline}}} \right) \, /{\text{ baseline}}$$

### ***Relative importance of multiple factors influencing the PCO***_***2***_***:CCO***_***2***_*** relationship during induction and resuscitation of endotoxemic shock***

Besides physically dissolved CO_2_ ([CO_2_]), which depends only on PCO_2_ under isothermic conditions, the two major factors influencing the PCO_2_:CCO_2_ relationship in the Douglas equation [37] are the HCO_3_^−^ factor (F_Bic_, i.e., the effect of pH on the quantity of [HCO_3_^−^] in both plasma and red blood cell) and the hemoglobin (Hb) factor (F_Hb_, i.e., the impact of Hb binding to CO_2_). Meanwhile, the F_Hb_ consists of three subfactors: the Hb concentration (F_Hb-Hb_), the SO_2_ (F_Hb-SO2_), and the pH (F_Hb-pH_) on Hb binding of CO_2_. The formulas for assessing the contribution of the two major factors and the three subfactors are provided in the supplemental material. After calculating the actual values of each factor and subfactor using these equations, the relative importance of the changes in each factor and subfactor was calculated at each measurement point. Thus, the influence of each specific factor or subfactor at any measurement point depends on how far its ratio differs from 1.00.

### Statistical analysis

Data were checked for normality using the Kolmogorov–Smirnov test and showed as means ± SD unless stated otherwise. Comparisons of data within groups were performed using repeated measures ANOVA or the Friedman test, as appropriate. Post-hoc paired *t*-tests or Wilcoxon signed-rank tests were used for one-time comparisons, as appropriate. The Bonferroni method was used to adjust for multiple comparisons. The relationships between variables were tested using the Spearman–Rho test, while a coefficient of determination (*R*^2^) was calculated to assess the strength of these associations. Statistical analysis was performed using GraphPad Prism 10.4.1 software for Windows (Boston, Massachusetts, USA) and SPSS statistical software package (IBM SPSS Statistics v. 25; IBM, Armonk, NY). A *p* < 0.0071 was considered statistically significant for the within-group (with the baseline) comparisons. All reported *P* values are two-sided.

## Results

### Systemic and regional splanchnic hemodynamics, microcirculatory flows, and oxygen-derived variables

Systemic and regional splanchnic hemodynamics, oxygen-derived parameters, microcirculatory blood flows (both by laser flowmetry, and direct video microscopy), volume of resuscitation fluids, and dose of vasopressors are shown in Table S1 and Figures S2 S6.

### Time course of venous and arterial pH

Arterial and mesenteric venous pH (i.e., pHa and pHvmes) followed similar trajectories (Table 1; Figure S7). Both pHa and pHvmes decreased significantly from the baseline to shock time (TS) and continued to decrease until their lowest values were reached at T2. Then, these slightly increased but remained significantly lower than their baseline values (Table 1; Figure S7). Arterial and mesenteric venous HCO_3_^−^ (Table 1; Figure S8) followed a similar patterns (Table 1; Figure S8).

**Table 1:** Arterial and mesenteric venous blood gases

	Hemoglobin (g/dL)	Arterial HCO_3_^−^ (mmol/L)	SaO_2_ (%)	PaCO_2_ (mmHg)	Arterial pH	Arterial lactate (mmol/L)	SvmesO_2_ (%)	Mesenteric venous pH	PvmesCO_2_ (mmHg)	Mesenteric venous Lactate (mmol/L)	Mesenteric venous HCO_3_^−^ (mmol/L)	ΔPCO_2_ (mmHg)
Baseline	11.4 ± 1.5	21.0 ± 3.2	100 ± 0.0	39.5 ± 5.7	7.49 ± 0.05	1.67 ± 0.43	63.1 ± 16.9	7.41 ± 0.05	48.2 ± 8.6	2.07 ± 0.83	26.7 ± 3.5	8.7 ± 4.2
Shock	12.7 ± 1.4^#^	15.9 ± 2.2^#^	99.3 ± 1.3	38.2 ± 6.2	7.41 ± 0.07^#^	3.87 ± 2.06^#^	37.2 ± 10.3^#^	7.36 ± 0.04^#^	60.0 ± 7.6^#^	4.75 ± 1.68^#^	21.9 ± 2.7^#^	21.8 ± 6.6^#^
T1	11.6 ± 1.4	16.3 ± 1.7^#^	97.1 ± 6.5	42.0 ± 6.2	7.36 ± 0.08^#^	3.77 ± 1.74^#^	52.3 ± 19.3	7.29 ± 0.08^#^	54.7 ± 8.8^#^	4.25 ± 1.73^#^	17.9 ± 2.6^#^	12.7 ± 5.5
T2	12.0 ± 1.7	15.0 ± 2.3^#^	97.7 ± 3.2	41.6 ± 8.2	7.34 ± 0.12^#^	3.99 ± 1.81^#^	50.1 ± 14.3^#^	7.26 ± 0.12^#^	56.1 ± 10.6^#^	4.32 ± 1.63^#^	16.9 ± 2.7^#^	14.5 ± 5.6^#^
T3	12.1 ± 2.1	14.8 ± 3.3^#^	98.0 ± 2.9	39.8 ± 5.7	7.34 ± 0.12^#^	3.87 ± 2.22^#^	51.7 ± 13.4	7.28 ± 0.10^#^	53.2 ± 8.4	4.11 ± 2.24	17.0 ± 3.3^#^	13.3 ± 6.6
T4	11.7 ± 1.6	16.3 ± 1.3^#^	99.1 ± 1.3	40.3 ± 5.9	7.37 ± 0.04^#^	2.63 ± 0.95	56.2 ± 14.7	7.33 ± 0.05^#^	50.4 ± 8.0	2.90 ± 0.89	18.4 ± 2.2^#^	10.2 ± 4.7
T5	11.4 ± 1.5	14.7 ± 1.2^#^	98.8 ± 1.4	41.4 ± 6.6	7.36 ± 0.02^#^	2.16 ± 0.54	57.2 ± 10.6	7.31 ± 0.05^#^	53.4 ± 7.0	2.53 ± 0.59	16.7 ± 1.9^#^	12.0 ± 4.6
T6	11.4 ± 1.6	14.6 ± 1.2^#^	98.7 ± 1.7	42.2 ± 7.7	7.36 ± 0.06^#^	1.87 ± 0.39	61.1 ± 8.3	7.31 ± 0.06^#^	53.2 ± 7.4	2.10 ± 0.58	16.2 ± 1.7^#^	11.0 ± 5.1

*HCO*_*3*_^*−*^ bicarbonate concentration, *SaO*_*2*_ arterial oxygen saturation, *PaCO*_*2*_ arterial carbon dioxide pressure, *SvmesO*_*2*_ mesenteric venous oxygen saturation, *PvmesCO*_*2*_ mesenteric venous carbon dioxide pressure, *ΔPCO*_*2*_, difference between mesenteric venous and arterial carbon dioxide pressures. Data are expressed as mean ± SD. ^#^p < 0.0071 vs. baseline. Data are presented as mean ± SD

### ***Time course of mesenteric venous SO***_***2***_***, arterial SO***_***2***_***, and lactate levels***

Arterial SO_2_ (SaO_2_) did not change significantly over time (Figure S9 and Table 1). However, SvmesO_2_ significantly decreased at TS (from 63.1 ± 16.9% at baseline to 37.2 ± 10.3% at shock, p = 0.0003) and then increased progressively from T1 until T6 with resuscitation (Table 1 and Figure S9). Table 1 and Figure S10 show the progression of arterial and mesenteric venous lactate levels.

### ***Time course of ΔPCO***_***2***_*** and ΔCCO***_***2***_

**Fig. 1:**
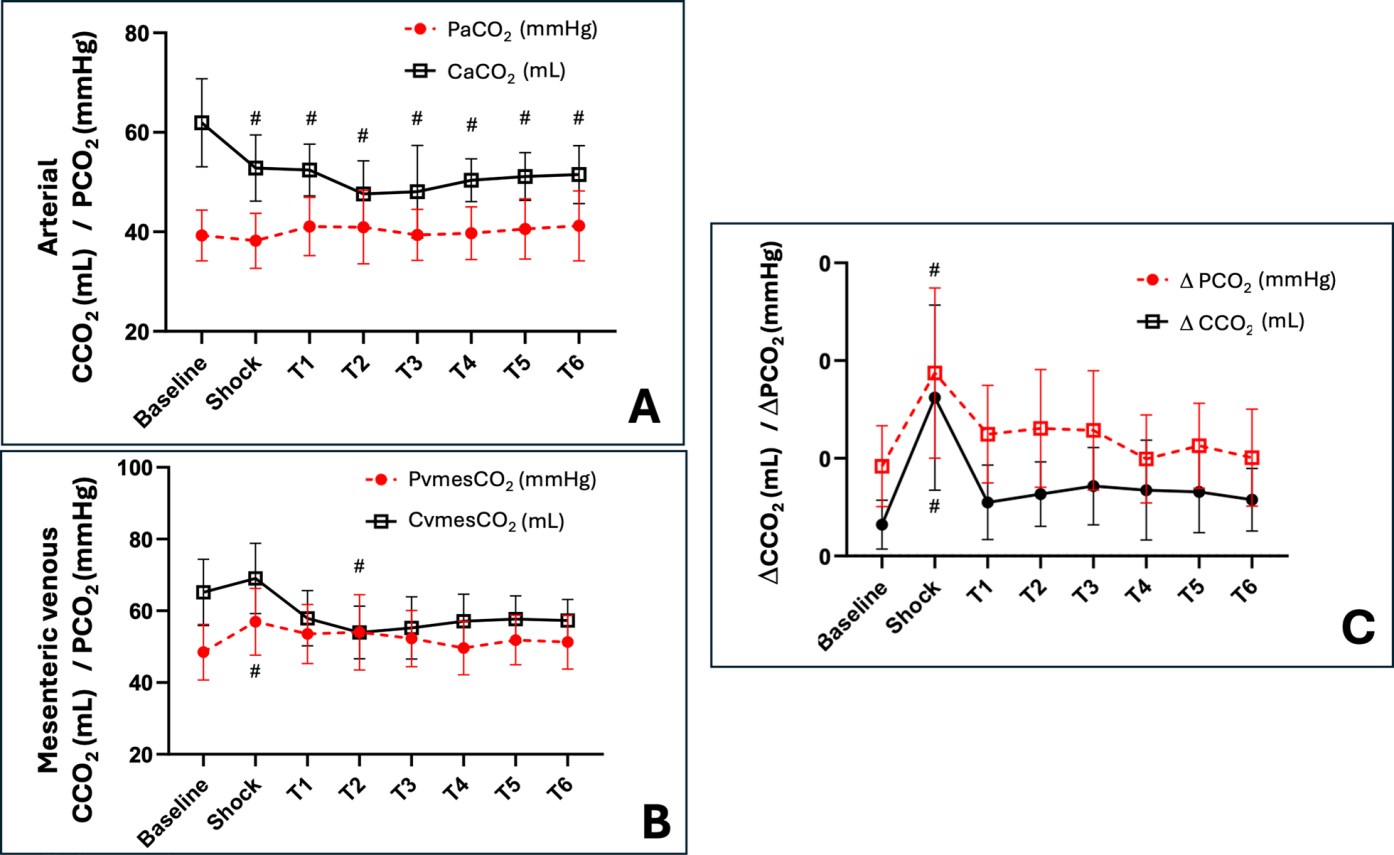
**A** Time course of arterial carbon dioxide pressure (PaCO_2_) and arterial carbon dioxide content (CaCO_2_). **B** Time course of mesenteric venous carbon dioxide pressure (PvmesCO_2_) and mesenteric venous carbon dioxide content (CvmesCO_2_). **C** Time course of the difference between arterial and mesenteric venous carbon dioxide pressures (ΔPCO_2_) and contents (ΔCCO_2_). #p < 0.0071 vs. baseline

ΔPCO_2_ and ΔCCO_2_ increased from baseline to TS as regional splanchnic and jejunal microvascular blood flows decreased (Figs. 1 Panel C; and Figures S2, S4, S5). Then, they decreased during the resuscitation period, paralleling the recovery of macro- and microvascular flow (Table 1; Figures S2, S4, S5). Remarkably, PaCO_2_ remained similar throughout the experiment, which suggests that the significant increase in ΔPCO_2_ from baseline until TS (8.7 ± 4.2 vs. 21.8 ± 6.6 mmHg, p < 0.0001) was mainly explained by the increase in mesenteric venous PCO_2_ (Fig. 1 Panels A, B; Table 1). Afterwards, ΔPCO_2_ decreased as mesenteric blood flow increased, until becoming not significantly different from its baseline value (p = 0.17) (Table 1; Fig. 1 Panel C). Conversely, mesenteric venous CCO_2_ did not increase significantly at TS from its baseline value (Table 2; Fig. 1 Panel B), while arterial CCO_2_ significantly decreased at TS and then remained significantly different from its baseline value (Table 2; Fig. 1: Panel A). Remarkably, even though ΔCCO_2_ and ΔPCO_2_ depicted similar trajectories (Table 2; Fig. 1: Panel C), the increase in ΔCCO_2_ at TS was mainly explained by CaCO_2_ decrease (i.e., more than CvmesCO_2_ increases).

**Table 2:** Comparisons between actual and default pH values and the percentage of disagreement in blood CO_2_ contents

	CvmesCO_2_ (mL)	CaCO_2_ (mL)	ΔCCO_2_ (mL)	Def_pHvmes_–CvmesCO_2_ (mL)	Def_pHa_–CaCO_2_ (mL)	Def_pH_–ΔCCO_2_ (mL)	Disagreement – CvmesCO_2_ (%)	Disagreement – CaCO_2_ (%)
Baseline	66.8 ± 9.9	63.7 ± 9.3	3.1 ± 2.9	66.8 ± 9.9	63.7 ± 9.3	3.1 ± 2.9	0	0
Shock	71.8 ± 8.6	51.7 ± 6.5^#^	20.1 ± 5.6^#^	82.7 ± 11.2*^#^	62.5 ± 10.7*	20.2 ± 11.9^#^	15.3 ± 10.8^#^	21.8 ± 22.1^#^
T1	56.9 ± 6.7^#^	51.3 ± 4.5^#^	5.6 ± 3.7	76.2 ± 12.7*	69.4 ± 9.8*	6.8 ± 9.2	39.8 ± 22.1^#^	40.0 ± 21.5^#^
T2	53.7 ± 7.6^#^	47.1 ± 6.7^#^	6.6 ± 3.3	77.4 ± 13.4*	68.2 ± 12.0*	9.2 ± 9.5	47.6 ± 41.9^#^	48.4 ± 39.8^#^
T3	53.5 ± 8.6^#^	45.9 ± 9.1^#^	7.6 ± 4.3	73.6 ± 13.7*	65.6 ± 11.3*	8.0 ± 10.4	40.8 ± 39.6^#^	50.4 ± 51.7^#^
T4	56.1 ± 7.5^#^	49.4 ± 3.6^#^	6.6 ± 5.6	70.6 ± 16.6*	67.0 ± 14.5*	3.6 ± 9.0	25.3 ± 20.2^#^	35.0 ± 23.9^#^
T5	56.8 ± 6.6	49.6 ± 3.8^#^	7.1 ± 4.5	74.6 ± 15.5*	68.8 ± 15.3*	5.8 ± 9.6	31.1 ± 20.8^#^	37.8 ± 24.6^#^
T6	56.9 ± 6.4	50.0 ± 5.1^#^	6.9 ± 2.7	74.5 ± 17.2*	70.3 ± 17.7*	4.1 ± 10.1	24.8 ± 21.3	29.0 ± 21.2^#^

*CvmesCO*_*2*_ mesenteric venous carbon dioxide content, *CaCO*_*2*_ arterial carbon dioxide content, *Def*_*pHvmes*_*–CvmesCO*_*2*_ default mesenteric venous carbon dioxide content by omitting the changes in mesenteric venous pH with Douglas equation, *Def*_*pHa*_*–CaCO*_*2*_ default arterial carbon dioxide content by omitting the changes in arterial pH with Douglas equation; *Def*_*pH*_*–ΔCCO*_*2*_ default difference between mesenteric venous carbon dioxide content and arterial carbon dioxide content by omitting the changes in mesenteric venous and arterial pH with Douglas equation; %Error = (default − actual)/actual value × 100. ^#^p < 0.0071 vs. baseline. ^*^p < 0.0071 significant difference Def_pH_ vs. actual. Data are presented as mean ± SD or median and interquartile [25–75]

### ***Time course of ΔPCO***_***2***_***:Ca-vO***_***2***_*** and ΔCCO***_***2***_***:Ca-vO***_***2***_*** ratios***

Both ΔPCO_2_:Ca-vO_2_ and ΔCCO_2_:Ca-vO_2_ ratios are shown in Figure S11. These depicted a significant increase at TS, followed by recovery during fluid resuscitation.

### ***pH effects on the PCO***_***2***_***:CCO***_***2***_*** relationship***

**Fig. 2:**
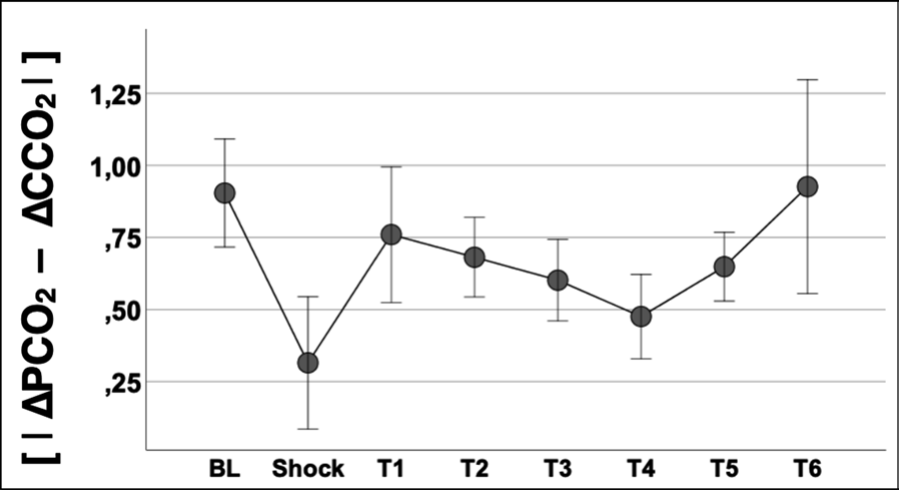
Time course of absolute ΔPCO_2_ to ΔCCO_2_ differences. |ΔPCO_2_| denotes the absolute arithmetic value of venous-to-arterial carbon dioxide partial pressures. |ΔCCO_2_| denotes the absolute arithmetic value of venous-to-arterial carbon dioxide contents. |ΔPCO_2_ – ΔPCO_2_| denotes the difference of absolute values of ΔPCO_2_ and ΔPCO_2_ (i.e., the absolute arithmetic distance between values)

**Fig. 3:**
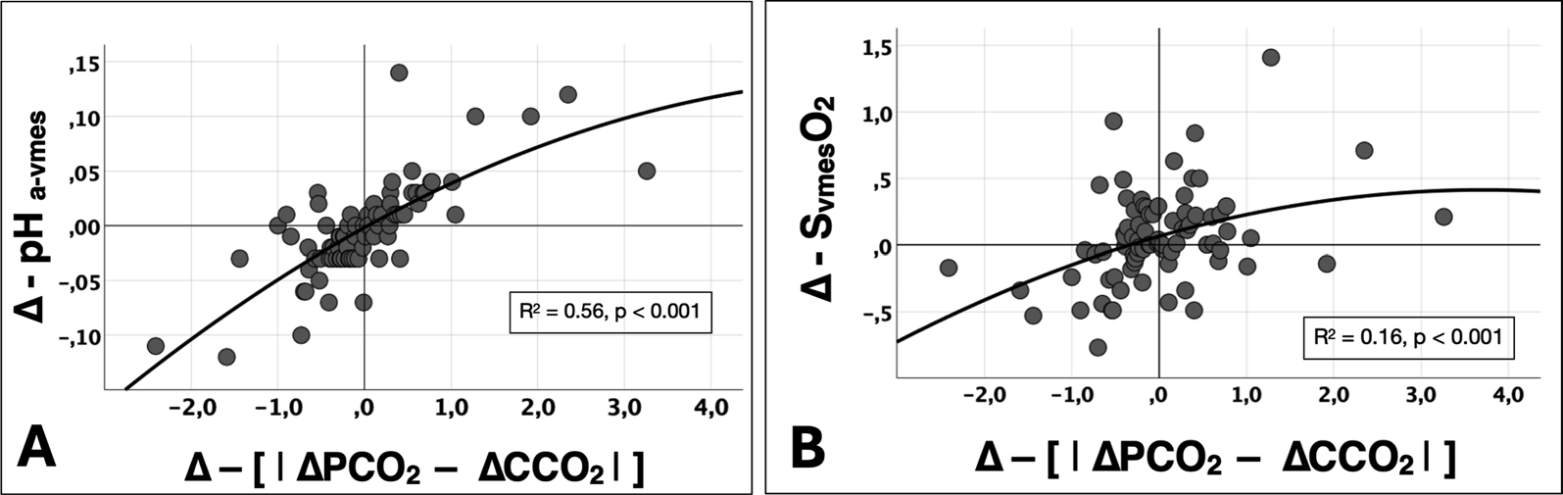
**A**. Relationship between variations in absolute arithmetic differences between ΔPCO_2_ and Δ CCO_2_ vs. variations in arterial-to-mesenteric pH (Δ–pH_a-vmes_), between adjacent time points; **B** relationship between variations in absolute arithmetic differences between ΔPCO_2_ and Δ CCO_2_ vs. variations in mesenteric venous oxygen saturation (Δ–S_vmes_O_2_), between adjacent time points. Δ – [|PCO_2_ – PCO_2_|] denotes the variation in the absolute arithmetic difference in ΔPCO_2_ and ΔCCO_2_ between adjacent time points. Δ–pH_a-vmes_ denotes the variation of arterial-to-venous pH difference between adjacent time points. Δ–S_vmes_O_2_ denotes the variation of arterial-to-venous pH difference between adjacent time points

The absolute arithmetic differences between ΔPCO_2_ and ΔCCO_2_ at every measurement time point (i.e., the [|ΔPCO_2_ – ΔCCO_2_|]) are depicted in Fig. 2. The [|ΔPCO_2_ – ΔCCO_2_|] decreased at TS while it returned to similar baseline values during the resuscitation period (Fig. 2). Variations in the absolute arithmetic ΔPCO_2_ to ΔCCO_2_ difference between adjacent measurement time points (i.e., the ∆–[|∆PCO_2_ – ∆CCO_2_|]) showed a good agreement with simultaneous variations in the arterial-to-mesenteric venous pH differences (Δ–pH_a-vmes_) (R^2^ = 0.56, p < 0.001) (Fig. 3, panel A), while they were poorly correlated with variations in mesenteric venous oxygen saturation (∆–S_vmes_O_2_) (R^2^ = 0.16, p < 0.001) (Fig. 3, panel B).

**Fig. 4:**
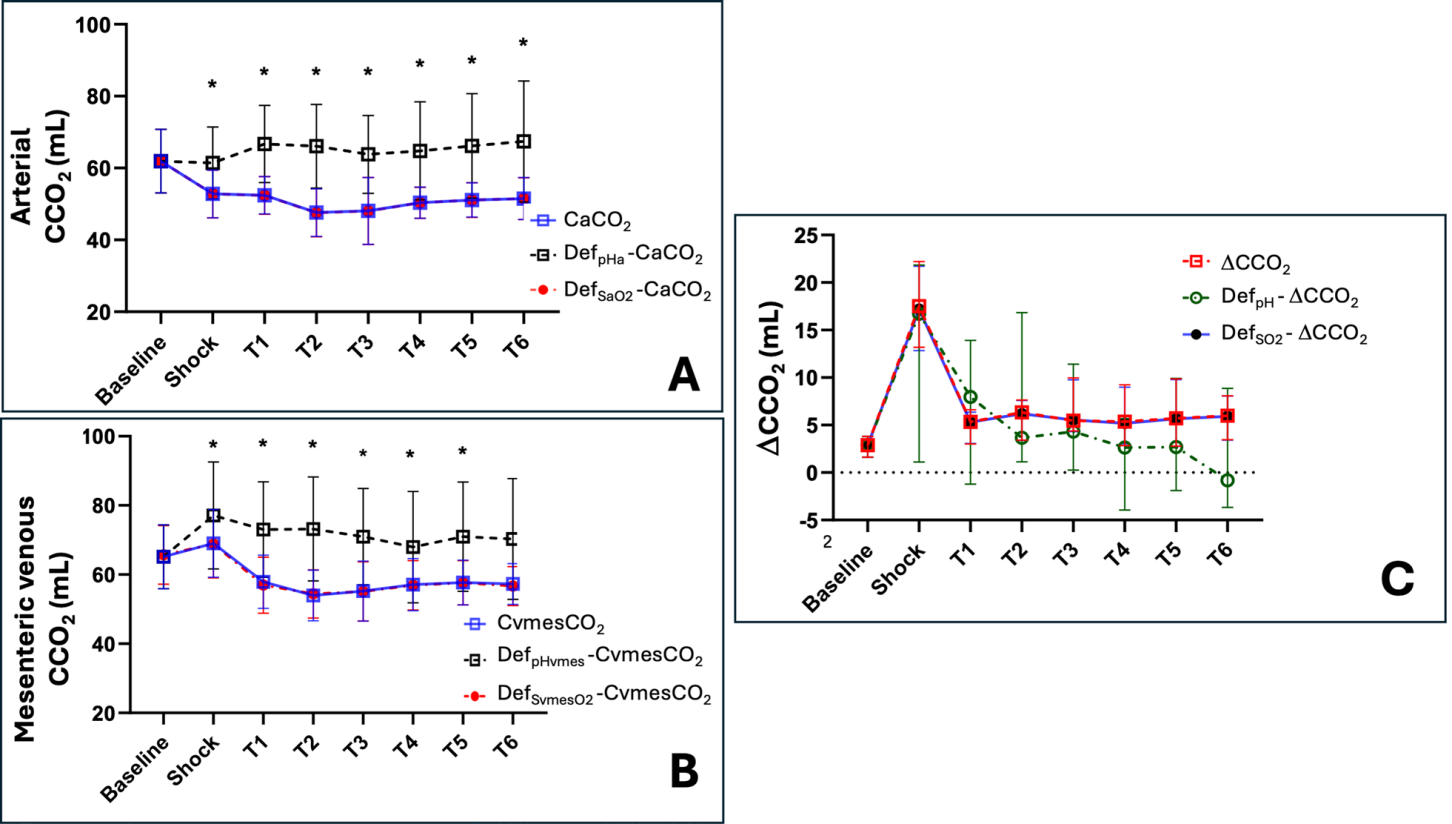
**A** Time course of arterial carbon dioxide content (CaCO_2_), default arterial pH arterial carbon dioxide content (Def_pHa_–CaCO_2_), and default arterial oxygen saturation arterial carbon dioxide content (Def_SaO2_–CaCO_2_). **B** Time course of mesenteric venous carbon dioxide content (CvmesCO_2_), default mesenteric venous pH mesenteric venous carbon dioxide content (Def_pHvmes_–CvmesCO_2_), and default mesenteric venous oxygen saturation mesenteric venous carbon dioxide content (Def_SvmesO2_–CvmesCO_2_). **C** Time course of the difference between arterial carbon dioxide content and mesenteric venous carbon dioxide content (ΔCCO_2_) and the default pH ΔCCO_2_ (Def_pH_–ΔCCO_2_) and default oxygen saturation ΔCCO_2_ (Def_SO2_–ΔCCO_2_). *p < 0.0071 significant difference Def_pH_ vs. actual

**Fig. 5:**
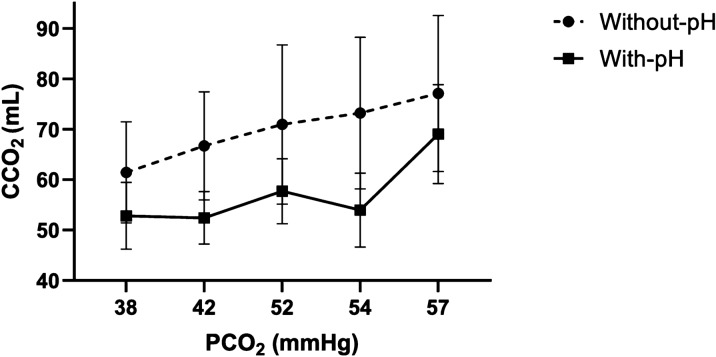
Carbon dioxide content (CCO_2_) as a function of carbon dioxide pressure (PCO_2_); CCO_2_ calculation by Douglas equation accounting for pH changes (with-pH) and without accounting for pH changes (without-pH)

The arterial and mesenteric venous CCO_2_ values showed significant differences with their respective original CCO_2_ values when recalculated without accounting for pH changes (i.e., Def_pHvmes_CvmesCO_2_ and Def_pHa_CaCO_2_) (Table 2 and Fig. 4). Both arterial and mesenteric venous PCO_2_ followed similar trajectories to Default_pH_ CCO_2_ during the experiment (Figures S12 and S13). Evolvement of ΔCCO_2_ and Def_pH_ΔCCO_2_ are detailed in Table 2. Remarkably, the significant increase in Def_pH_ΔCCO_2_ at the time of shock was more influenced by mesenteric venous than arterial CCO_2_ variations (Table 2; Figures S12 and S13). On the other hand, the relationship between PCO_2_ and CCO_2_ was almost linear when omitting changes in pH, while it became non-linear when such changes were acknowledged (Fig. 5). A summary of % disagreement between mesenteric venous CCO_2_ and their respective Default pH values is shown in Table S2.

### ***SO***_***2***_*** effects (Haldane) on the PCO***_***2***_***:CCO***_***2***_*** relationship***

There was a poor correlation between the variations in SvmesO_2_ (Δ–S_vmes_O_2_) and the ∆–[|∆PCO_2_ – ∆CCO_2_|] (R^2^ = 0.16, p < 0.001) (Fig. 3 Panel B). Similarly, there were no significant differences between mesenteric venous CCO_2_ and their respective Default-SvO_2_ values over time (Table 3; Fig. 4 Panel B), as also occurred in the arterial side (Fig. 4 Panel A). Furthermore, ΔCCO_2_ values evolved similarly to their respective Default-SO_2_ CCO_2_ (Fig. 4 Panel C; Table 3). In addition, ΔPCO_2_ and ΔCCO_2_ not accounting for SO_2_ (Def_SO2_ ΔCCO_2_) evolved parallelly over time (Figure S14, S15). A summary of % disagreement between mesenteric venous CCO_2_ and their respective Default-SvO_2_ values is shown in Table S2.

**Table 3:** Comparisons between actual and default oxygen saturation values and the percentage of disagreement in blood CO_2_ contents

	CvmesCO_2_ (mL)	CaCO_2_ (mL)	ΔCCO_2_ (mL)	Def_SvmesO2_–CvmesCO_2_ (mL)	Def_SaO2_–CaCO_2_ (mL)	Def_SO2_–ΔCCO_2_ (mL)	Disagreement – CvmesCO_2_ (%)	Disagreement – CaCO_2_ (%)
Baseline	66.8 ± 9.9	63.7 ± 9.3	3.1 ± 2.9	66.8 ± 9.9	63.7 ± 9.3	3.1 ± 2.9	0	0
Shock	71.8 ± 8.6	51.7 ± 6.5^#^	20.1 ± 5.6^#^	71.5 ± 8.6	51.7 ± 6.5^#^	19.8 ± 5.5^#^	− 0.39 ± 0.27^#^	− 0.01 ± 0.02
T1	56.9 ± 6.7^#^	51.3 ± 4.5^#^	5.6 ± 3.7	56.8 ± 6.7^#^	51.3 ± 4.5^#^	5.5 ± 3.6	− 0.16 ± 0.26	− 0.05 ± 0.13
T2	53.7 ± 7.6^#^	47.1 ± 6.7^#^	6.6 ± 3.3	53.6 ± 7.6^#^	47.1 ± 6.7^#^	6.5 ± 3.3	− 0.19 ± 0.20	− 0.04 ± 0.07
T3	53.5 ± 8.6^#^	45.9 ± 9.1^#^	7.6 ± 4.3	53.4 ± 8.5^#^	45.9 ± 9.1^#^	7.5 ± 4.2	− 0.16 ± 0.25	− 0.03 ± 0.05
T4	56.1 ± 7.5^#^	49.4 ± 3.6^#^	6.6 ± 5.6	56.0 ± 7.4^#^	49.4 ± 3.6^#^	6.5 ± 5.4	− 0.13 ± 0.29	− 0.01 ± 0.02
T5	56.8 ± 6.6	49.6 ± 3.8^#^	7.1 ± 4.5	56.7 ± 6.5	49.6 ± 3.8^#^	7.1 ± 4.4	− 0.11 ± 0.26	− 0.02 ± 0.02
T6	56.9 ± 6.4	50.0 ± 5.1^#^	6.9 ± 2.7	56.8 ± 6.4	50.0 ± 5.1^#^	6.8 ± 2.7	− 0.12 ± 0.20	− 0.02 ± 0.02

*CvmesCO*_*2*_ mesenteric venous carbon dioxide content, *CaCO*_*2*_ arterial carbon dioxide content, *Def*_*SvmesO2*_*–CvmesCO*_*2*_ default mesenteric venous carbon dioxide content by omitting the changes in mesenteric venous oxygen saturation with Douglas equation; *Def*_*SaO2*_*–CaCO*_*2*_ default arterial carbon dioxide content by omitting the changes in arterial oxygen saturation with Douglas equation; *Def*_*SO2*_*–ΔCCO*_*2*_ default difference between mesenteric venous carbon dioxide content and arterial carbon dioxide content by omitting the changes in mesenteric venous and arterial oxygen saturation with Douglas equation; %Error = (default − actual)/actual value × 100. ^#^p < 0.0071 vs. baseline. ^*^p < 0.0071 significant difference Def_pH_ vs. actual. Data are presented as mean ± SD or median and interquartile [25–75]

### ***Relative importance of multiple factors on the PCO***_***2***_***:CCO***_***2***_*** relationship***

The influence of each of several factors on the PCO_2_:CCO_2_ relationship is shown in Table S3, with “factor influence” increasing as its value deviates from 1.0. Thus, deviations of F_bic_ (which are due to pH changes) during induction and resuscitation of this endotoxemic shock model far exceed those of F_Hb_ (Table S3). Considering the subfactors of F_Hb_, the deviations of F_Hb-pH_ (i.e., because of the effect of pH changes on [NH–CO_2_]) exceed those of F_Hb-SO2_ and F_Hb-Hb_, both in venous and arterial blood. Thus, the changes and influence of pH (F_bic_ and F_Hb-pH_) were quantitatively more important in blood CO_2_ transport (both in venous and arterial blood), than those provoked by Hb and SO_2_.

## Discussion

This study reveals some important findings: (1) regional splanchnic PCO_2_:CCO_2_ relationship varies dynamically over time under the changing conditions of development and resuscitation of endotoxemic shock; (2) dynamic variations in the absolute arithmetic ΔPCO_2_ to ΔCCO_2_ difference (i.e., the ∆–[|PCO_2_ – CCO_2_|]) showed a good agreement with variations in arterial-to-venous pH differences (Δ–pH_a-vmes_), highlighting the key influence of hydrogen ion accumulation on PCO_2_:CCO_2_ relationship during endotoxemic shock conditions; (3) PCO_2_:CCO_2_ relationship was less influenced by variations in SvmesO_2_ (Haldane effect), in the range observed in this experiment; (4) recalculating CCO_2_ values by ignoring changes in pH (Def_pH_) or SO_2_ (Def_SO2_) suggests in favor of the predominant influence of pH variations over the Haldane effect on the PCO_2_:CCO_2_ dissociation curve; (5) the increase in ΔPCO_2_ at the time of shock was mainly due to the increase in PvmesCO_2_, while the simultaneous increase in ΔCCO_2_ was mainly explained by the predominant decrease in CaCO_2_, which is in line with observations in healthy humans exceeding the lactate threshold during exhausting exercise.

CO_2_ is a terminal product from aerobic and anaerobic biochemical processes of cell metabolism. About 85% of CO_2_ is carried in blood as bicarbonate, 5% dissolved in plasma, and the other 5% in combination with proteins as carbamino compounds [38]. These last occur when CO_2_ combines with terminal amine groups in blood proteins, especially with the globin chains of hemoglobin [38]. Hemoglobin-O_2_ saturation (SO_2_) is a major factor affecting the capacity of hemoglobin to fix CO_2,_ a phenomenon known as the Haldane effect [38, 39], so that CCO_2_ increases when blood is deoxygenated, while CCO_2_ diminishes when blood is oxygenated at any assumed PCO_2_.

Studies investigating the impact of the Haldane effect on the PCO_2_:CCO_2_ relationship in critically ill patients are limited. Jakob et al. [34] suggested that paradoxical increases in gastric mucosal PCO_2_ gradient during increasing splanchnic blood flow in post-cardiac surgery patients could be explained by the Haldane effect. They computed the mucosal-to-arterial CCO_2_ according to Giovannini et al. [3], estimated the influence of the Haldane effect in gastric mucosa by calculating CCO_2_ acknowledging or not oxygen consumption (i.e., using default SO_2_ values), and assumed equal proportional changes in total splanchnic and mucosal perfusion and CO_2_ production. Unfortunately, gastric mucosal oxygen saturation was not measured but estimated from the gastric mucosal oxygen extraction, and only pairs of measurements were obtained (before and after dobutamine infusion), which do not accurately reflect the intricate PCO_2_:CCO_2_ relationship that occurs during the dynamic conditions of the development and resuscitation of septic shock.

The PCO_2_:CCO_2_ relationship is not static. Indeed, it varies dynamically over time as environmental conditions change, such as occurred in our experimental model of induction and resuscitation of endotoxemic shock (Fig. 2). To determine the influence of the Haldane effect vs. hydrogen ion accumulation on the dynamic PCO_2_:CCO_2_ relationship, we used two approaches: first, we calculated the absolute arithmetic ΔPCO_2_ to ΔCCO_2_ difference (i.e., the [|ΔPCO_2_ – ΔCCO_2_|]) at every measurement time point (Fig. 2); then, we calculated the magnitude of such discrepancies between pairs of adjacent time-point measurements (from baseline till shock, from shock till T1, T1 till T2, and so on), i.e., the ∆–[|ΔPCO_2_ – ΔCCO_2_|]. Afterwards, ∆–[|ΔPCO_2_ – ΔCCO_2_|] were plotted against dynamic variations in SmesO_2_ (∆–S_vmes_O_2_) (Fig. 3, panel B) and against variations in arterial-to-venous pH (∆–pH_a-vmes_) (Fig. 3, panel A); and second, we recalculated the time course of arterial and venous CCO_2_ values maintaining baseline pH (Def_pH_) or SO_2_ values (Def_SO2_). Using these two approaches, the Haldane effect had no major influence on the PCO_2_:CCO_2_ relationship as suggested by the almost invariable behavior of CCO_2_ when ignoring or acknowledging SvmesO_2_ (Table 3 and Fig. 4) and by the poor correlation between ∆–[|ΔPCO_2_ – ΔCCO_2_|] and ∆–S_vmes_O_2_ (Fig. 3, Panel B). These results are in line with Sun et al. [26], who found that changes in SO_2_ (Haldane effect) had a minor influence on the PCO_2_:CCO_2_ relationship (CCO_2_ calculated using the Douglas equation) in healthy volunteers subjected to heavy exercise. Similar findings were reported by Mesquida et al. [31] in patients with septic shock and by Mallat et al. [40] in a vascularly isolated hindlimb of a dog model, both suggesting a limited influence of the Haldane effect on the CO_2_ dissociation curve.

Although it is known that metabolic acidosis can also affect the PCO_2_:CCO_2_ relationship [1], the extent to which hydrogen ion accumulation influences CCO_2_ under hypoperfusion and tissue hypoxia conditions has not been widely described. In this regard, our data suggest a good correlation between the dynamic variations in PCO_2_:CCO_2_ relationship (quantified as ∆–[|PCO_2_ – CCO_2_|]) vs. dynamic changes in pH (∆–pH_a-vmes_) (Fig. 3, Panel A), thus suggesting the key influence of hydrogen ion accumulation (as result of non-aerobic CO_2_ generation during shock conditions) on the PCO_2_:CCO_2_ dissociation curve. In addition, mesenteric venous and arterial CCO_2_ differed significantly from their respective original CCO_2_ when pH values were not acknowledged (Table 3 and Fig. 4). Remarkably, when changes in pH were not allowed for calculations, the PCO_2_:CCO_2_ relationship was almost parallel (Figures S12, S13), while CCO_2_ and PCO_2_ depicted different trajectories when changes in pH were acknowledged (Fig. 5). These findings agree with previous observations suggesting a significant influence of pH variations on the PCO_2_:CCO_2_ dissociation curve, causing CCO_2_ to become not linearly related to PCO_2_. Interestingly, ∆CCO_2_ became high at TS in our model because of a simultaneous decreasing arterial plus increasing venous CCO_2_. In addition, the pH factor (F_bic_ from 1.00 at baseline) even dominated the PCO_2_:CCO_2_ relationship over changes in Hb concentration and SO_2_ (Table S2). Consequently, when the CCO_2_ is calculated from PCO_2_, changes in blood pH must not be ignored. In line with our results, other observations suggest the predominant role of hydrogen ion accumulation on the PCO_2_:CCO_2_ dissociation curve in patients with septic shock [31]. Similarly, an experimental model also demonstrated that hydrogen ion accumulation is related to a non-linear association between PvCO_2_ and CvCO_2_ during controlled conditions of ischemic and hypoxic hypoxia [40].

Interestingly, results about ∆CCO_2_ were different: ΔCCO_2_ values did not differ from their respective Default pH-ΔCCO_2_ calculations (Table 2 and Fig. 4, panel C). In this regard, Def_pH_ΔCCO_2_ and ΔPCO_2_ had the same progression patterns over time (Figure S14), which suggests that CvmesCO_2_ and CaCO_2_ evolved in opposite directions during shock and consequently were to be offset in calculating ΔCCO_2_. Similar findings were described during exhaustion exercise in which arterial and venous CCO_2_ evolved discordantly after reaching the apparent “lactic acidosis threshold” [33].

In our model, ΔPCO_2_ decreased as regional mesenteric and microvascular blood flows were restored. Thus, the progression of ΔPCO_2_ tracking variations in regional splanchnic and jejunal microvascular blood flows, and the predominant influence of hydrogen ion concentration (tissue acidosis) over the Haldane effect, might support the reliability of ΔPCO_2_ (and its combination with Ca-vO_2_) as a marker of tissue perfusion and cell metabolic dysfunction during the evolvement and recovery of vasodilated shock. While ΔPCO_2_ has been related to both macro- and microvascular blood flow, ΔPCO_2_:Ca-vO_2_ and ΔCCO_2_:Ca-vO_2_ ratios have been suggested as markers of tissue oxygenation. Indeed, elevated ΔPCO_2_:Ca-vO_2_ values might predict significant increases in global oxygen consumption after volume expansion [32], while other markers did not. Nevertheless, abnormal ΔPCO_2_:Ca-vO_2_ values have been linked to the Haldane effect, thus challenging its utility as a marker of tissue perfusion during resuscitation of shock. However, our results suggest the predominance of hydrogen ion accumulation over the Haldane effect on the PCO_2_:CCO_2_, which reinforces the idea of ΔPCO_2_:Ca-vO_2_ and ΔCCO_2_:Ca-vO_2_ ratios as markers of tissue perfusion and altered cell metabolism. Thus, elevations of ΔPCO_2_:Ca-vO_2_ and ΔCCO_2_:Ca-vO_2_ ratios suggest an extra generation of hydrogen ions finally contributing to non-aerobic CO_2_ production. Thus, ΔPCO_2_:Ca-vO_2_ and ΔCCO_2_:Ca-vO_2_ ratios should not be seen as a simple consequence of the Haldane effect, but instead as markers of altered cell metabolism during shock, and consequently, they could be used along with other perfusion markers to interpret the dynamic process of reperfusion during resuscitation of shock.

We admit important limitations in our study. First, it was a secondary analysis of a previous experimental endotoxemic shock model. Nevertheless, general conditions, blood sampling, and resuscitation protocol were systematically reproduced. Second, the computation of CCO_2_ is subject to an important risk of errors due to the number of variables included in the equation [29], which might amplify the subsequent calculation of ΔCCO_2_. Third, formulas used to calculate CCO_2_ have been derived from data coming from healthy humans during resting and near steady-state exercise conditions, which might limit its applicability to those dynamically variable conditions observed during induction and resuscitation of vasodilated shock in which the time needed to equilibrate different CO_2_ stores (i.e., CO_2_ dissolved in plasma, bicarbonate, and CO_2_ in tissues) might be highly variable. Fourth, in general, the magnitude of regional variations in SvmesO_2_ was relatively small, which could limit the influence of the Haldane effect. Nevertheless, the extent of SvmesO_2_ decrease from baseline till the time of shock was enough to deduce the predominant effect of hydrogen ion accumulation on the PCO_2_:CCO_2_ relationship.

## Conclusions

During induction and resuscitation of endotoxemic shock, discrepancies between carbon dioxide pressures (PCO_2_) and contents (CCO_2_) are mostly explained by hydrogen ion accumulation rather than the Haldane effect. The predominant influence of regional acidosis on PCO_2_:CCO_2_ dissociation curve could have profound implications when interpreting ΔPCO_2_ and its combination with arterial-to-venous oxygen differences during resuscitation of vasodilated shock.

## Supplementary Information

Supplementary file 1.

## Data Availability

Data types: complete experimental data (but these will not be available through repositories or websites). How to access data: request must be sent to Prof. Gustavo A. Ospina-Tascón (gusospin@gmail.com). When available: after main manuscript publication. Additional information who can access the data: researchers interested in using data for prespecified scientific purposes. Supporting document types: official letter indicating how data will be used. Mechanisms of data availability: data should be available after evaluation and approval by the sponsor and the trial steering committee.

## Abbreviations

CO_2_: Carbon dioxide
∆PCO_2_: Venous-to-arterial carbon dioxide tension difference
CCO_2_: CO_2_ content
