# Supplementary material for: Regional venous-to-arterial carbon dioxide pressure and content differences during endotoxemic shock: influence of hydrogen ion accumulation vs. Haldane effect
Source: Intensive Care Med Exp. 2025 Sep 8;13:93. doi: 10.1186/s40635-025-00805-0 (PMC12417345; doi:10.1186/s40635-025-00805-0)
Supplement: Supplementary file 1 [file 40635_2025_805_MOESM1_ESM.pdf]

Regional venous–to–arterial carbon dioxide pressure and content differences during endotoxemic shock: influence of hydrogen ion accumulation vs. Haldane effect

Supplemental Material

Gustavo A. Ospina-Tascón MD, PhD [1,2]; Daniel De Backer MD, PhD [3]; José L. Aldana MD, MSc [1,2]; Alberto F. García Marín MD, MSc [1, 2]; Luis E. Calderón MD [1,2]; Gustavo García-Gallardo MD [1,2]; Nicolás Orozco MD [1,2]; Jihad Mallat MD, PhD [4, 5, 6]

1. Department of Intensive Care, Fundación Valle del Lili. Cali, Colombia
2. Translational Research Laboratory in Critical Care Medicine (TransLab-CCM), Universidad Icesi, Cali, Colombia.
3. Intensive Care Department. CHIREC Hospitals, Université Libre de Bruxelles, Brussels, Belgium
4. Critical Care Division, Integrated Hospital Care Institute, Cleveland Clinic Abu Dhabi. Abu Dhabi, United Arab Emirates.
5. Cleveland Clinic Lerner College of Medicine of Case Western Reserve University, Cleveland, OH, USA.
6. Faculty of Medicine, University of Banja Luka. Banja Luka, Republic of Srpska, Bosnia and Herzegovina.

Address for correspondence:

Gustavo A. Ospina-Tascón, M.D., Ph.D.

Department of Intensive Care, Fundación Valle del Lili

Translational Laboratory in Critical Care Medicine (TransLab-CCM), Universidad Icesi

Cali, Colombia

Tel (+57) 602 331 9090 – Fax (+57) 602 331 9090 Ext: 8200

Email: gusospin@gmail.com; gaospina@icesi.edu.co

TABLE OF CONTENTS

SUPPLEMENTARY MATERIALS AND METHODS 3

ANIMAL PREPARATION AND ANESTHESIA 3
SURGICAL PREPARATION / MONITORING INSTALLATION 3
GENERAL MONITORING 5
EXPERIMENTAL PROTOCOL 6
CALCULATING THE FACTORS INFLUENCING THE PCO2:CCO2 RELATIONSHIP 7
MICROCIRCULATORY MEASUREMENTS 8

SUPPLEMENTARY FIGURES 10

FIGURE S1. EXPERIMENTAL TIMELINE. 10
FIGURE S2. TIME-COURSE OF SYSTEMIC AND SPLANCHNIC HEMODYNAMICS 11
FIGURE S3. TIME-COURSE OF SPLANCHNIC AND SYSTEMIC OXYGEN-DERIVED PARAMETERS 12
FIGURE S4. TIME-COURSE OF JEJUNAL MICROCIRCULATORY BLOOD FLOWS 13
FIGURE S6. TIME-COURSE OF RESUSCITATION FLUIDS 15
FIGURE S7. TIME COURSE OF ARTERIAL AND MESENTERIC VENOUS PH 16
FIGURE S8. TIME COURSE OF ARTERIAL AND MESENTERIC VENOUS BICARBONATE (HCO_3^-)..................16
FIGURE S9. TIME COURSE OF ARTERIAL AND MESENTERIC VENOUS OXYGEN SATURATION (SO_2). 17
FIGURE S10. TIME COURSE OF ARTERIAL AND MESENTERIC VENOUS LACTATE LEVELS. 17
FIGURE S11. TIME-COURSE OF $\Delta PCO_2 : \Delta PO_2$ AND $\Delta PCO_2 : \Delta PO_2$ RATIOS. 18
FIGURE S12. TIME COURSES OF ARTERIAL CARBON DIOXIDE PRESSURE ($PACO_2$) AND DEFAULT ARTERIAL PH ARTERIAL - CARBON DIOXIDE CONTENT 19
FIGURE S13. TIME COURSES OF MESENTERIC VENOUS CARBON DIOXIDE PRESSURE ($PVMESCO_2$) AND DEFAULT MESENTERIC VENOUS PH MESENTERIC VENOUS CARBON DIOXIDE CONTENT 19
FIGURE S14. TIME COURSES OF THE DIFFERENCE BETWEEN MESENTERIC VENOUS CARBON DIOXIDE PRESSURE AND ARTERIAL CARBON DIOXIDE PRESSURE (ΔPCO_2) AND DEFAULT PH DIFFERENCE BETWEEN MESENTERIC VENOUS CARBON DIOXIDE CONTENT AND ARTERIAL CARBON DIOXIDE CONTENT 20
FIGURE S15. TIME COURSES OF MESENTERIC VENOUS CARBON DIOXIDE PRESSURE ($PVCO_2$) AND DEFAULT MESENTERIC VENOUS OXYGEN SATURATION MESENTERIC VENOUS CARBON DIOXIDE CONTENT 20

SUPPLEMENTARY TABLES 21

TABLE S1. HEMODYNAMICS AND OXYGEN-DERIVED PARAMETERS 21
TABLE S2. COMPARISONS BETWEEN PERCENTUAL DISAGREEMENT BY NOT ACCOUNTING FOR MESENTERIC-VENOUS PH AND OXYGEN SATURATION VARIATIONS 27
TABLE S3. FACTORS DESCRIBING THE MAGNITUDE BY WHICH HB, SO_2, OR PH VARIATIONS AFFECT THE PCO_2:CCO_2 RELATIONSHIP 28

SUPPLEMENTARY REFERENCES..................29

Supplementary Materials and Methods

Animal preparation and anesthesia

The present study was approved by the institutional Animal Research Committee (CIECUAE 0021/2019). Twelve female Landrace pigs (32–38 kg) were kept fasting for a 12-h period, with free access to water. After a preconditioning period of at least 24 hours, animals were sedated with intramuscular doses of ketamine (5–10 mg/kg) and xylazine (0.1 mg/kg). Afterward, a venous access was inserted in the ear (Insyte Autoguard, Infusion therapy system; Sandy, Utah, USA) to ensure administration of sedative agents. Initial intravenous sedation was provided with a combination of propofol (2 – 4 mg·kg^{-1}), and fentanyl (2 – 5 µgr·kg^{-1}), and an endotracheal tube was placed while remaining in ventral position. Then, animals were positioned in supine position and connected to mechanical ventilation (Dräger Fabius plus XL anaesthesia machine; Lübeck, Germany) in assist control mode, setting a tidal volume of 12 ml·Kg^{-1} and adjusting minute ventilation to maintain arterial PCO_2 at 36 – 42 mmHg. A side-stream capnometer (Dräger, Scio four plus gas measurements module; Lübeck, Germany) was connected to the expiratory branch from the ventilator circuit. Total intravenous anesthesia was provided with midazolam (3 – 5 µgr·Kg^{-1}·min^{-1}), fentanyl (0.03 – 0.05 µgr·Kg^{-1}·min^{-1}), and propofol (50 µgr·Kg^{-1}·min^{-1}), while muscular paralysis was ensured with vecuronium bromide (5 µgr·Kg^{-1}·min^{-1}) throughout the entire experiment.

Surgical preparation / monitoring installation

Neck vessels were accessed by surgical dissection, and catheters were inserted in the aorta through carotid artery (Bi-lumen central venous 7-Fr catheter; CV-17702. Arrow International, Reading, PA. USA) to monitor aortic arterial pressure and to enable blood sampling for gas analyses and lactate measurements. The left internal jugular vein was dissected to be used for fast administration of fluids during the resuscitation phase, while a three-lumen catheter (Three-lumen central venous 7-Fr catheter; CV-25703. Arrow International Reading, PA. USA) was inserted through external right jugular vein to

continuously measure central venous pressure and for infusion of norepinephrine and endotoxin. A continuous-cardiac-output (CCO) pulmonary artery catheter (7.5-Fr, Edwards Swan-Ganz CCO; Baxter Edwards Critical Care. Irvine, CA. USA) was inserted through the right internal jugular vein to measure pulmonary arterial pressure, pulmonary artery occlusion pressure, and to withdraw mixed-venous blood samples. In addition, a thermistor-tipped catheter was inserted through right femoral artery and connected to a transpulmonary – thermodilution cardiac output monitoring system (PulsioFlex - PiCCO; PULSION Medical Systems AG; Münich, Germany) to monitor cardiac output, stroke volume, pulse pressure and stroke volume variations, and to estimate extra-vascular lung water, end-diastolic global volume, and pulmonary vascular permeability. Core temperature was continuously monitored using a thermistor at the tip of the femoral catheter (PulsioFlex - PiCCO; PULSION Medical Systems AG; Münich, Germany). External heating or cooling was used to maintain a central temperature of 36.5 ± 1.0 °C. Continuous electrocardiographic, pulsioximetry, and invasive pressures were recorded throughout the entire experiment (Drägger Infinity Vista XL; Drägger Medical System, Lübeck, Germany). Animals received intravenous lactate Ringer fixed infusion at 3 ml/kg during this surgical preparation phase. Unexpected loses in this period were compensated according to decision of the investigator team.

A midline laparotomy was performed, and abdominal dissection was completed up to expose the abdominal aorta in its supra celiac portion and the superior mesenteric artery. Immediately, ultrasound doppler flow probes (Transonic Systems Inc., Ithaca NY., USA) were placed around these two vessels (supra-celiac abdominal aorta and mesenteric arteries) and connected to ultrasound flowmeter modules (Transonic perivascular flow module TS420; Transonic Systems Inc., Ithaca NY., USA). Through a small ostomy in the antimesenteric jejunal wall, a small laser doppler (LDF) probe was carefully attached to the mucosa, while a second LDF probe was fixed to the jejunal serosa (OxyFlo Pro. Oxford Optronix, UK). Both ultrasound flowmeter and laser doppler signals were continuously recorded in a laptop (HP ProBook 440 G4. Hewlett Packard Development Company, LP; Palo Alto, CA. USA) by using a data acquisition system (PowerLab 4/35; Ad Instruments. Oxford, UK).

A double-lumen catheter (2-lumen central venous 4-Fr bi-lumen catheter; CS-14402. Arrow International. Morrisville, NC. USA) was inserted through the splenic vein up to the confluence with the superior mesenteric vein. Then, splenectomy was performed after arterial local constriction with epinephrine. An infusion with dextrose 5% at 5 ml/h was provided through this catheter to ensure its permeability during the experiment. A surgical cystostomy was also created and an air-balloon catheter inserted and fixed to the bladder to quantify urinary output. A jejunum loop was exteriorized through the midline incision, and a small segment was opened along its antimesenteric border using electrocautery. After careful hemostasis, the abdominal contents were returned to the cavity, and the abdomen was partially closed, leaving out the jejunostomy loop, which was then covered with moistened compresses and an anti-adherent bag to avoid heat and fluid loss. Such a loop was used to evaluate microcirculatory blood flow at jejunal mucosa at the pre-established time-points. Cables from ultrasound flowmeters and laser doppler probes were exteriorized throughout the midline incision.

General monitoring

Arterial pressure was recorded simultaneously at aortic arch and femoral artery during the entire experiment (Drägger Infinity Vista XL; Drägger Medical System, Lübeck, Germany). Cardiac output was measured by transpulmonary thermodilution (PulsioFlex - PiCCO; PULSION Medical Systems AG; Münich, Germany). Calibration of the system was performed each hour during the entire experiment by series of 10 mL boluses of normal saline 0.9% solution at 4 – 6 ºC injected through jugular central venous catheter. The average of three values of cardiac output obtained by such transpulmonary thermodilution was then recorded and calibrated with pulse contour (PulsioFlex - PiCCO; PULSION Medical Systems AG; Münich, Germany) to subsequently monitor cardiac output, stroke volume, and pulse pressure variations. Other transpulmonary thermodilution-derived variables recorded during the experiment included: systemic vascular resistance index (SVRI), global end-diastolic volume index (GEDVI), extravascular lung water index (EVLWI), and the pulmonary vascular permeability index (PVPI). Meanwhile, pulmonary artery catheter was used to

monitor mean pulmonary artery, central venous, and pulmonary arterial occlusion pressures, which were measured at the end of expiration and referenced to the mid-chest level. Pulmonary artery pressure was continuously recorded and tightly monitored during endotoxin infusion and the rest of the experiment.

Experimental Protocol

The timeline of the experiment is depicted in Figure S1. A stabilization period of 60 min was ensured after surgical preparation, catheter placement, and monitoring installation. Baseline measurements (BL) were then performed and endotoxic shock was induced by intravenous infusion of lipopolysaccharide (Escherichia coli O55:B5 purified by gel-filtration chromatography; Sigma-Aldrich; Saint Louis, MO. USA), starting at 0.5 μgr·kg-1·min^{-1} and escalating progressively until 6 μgr·kg^{-1}·min^{-1} [1] (over around 4 hours) and maintained for 30 minutes after fulfilling shock criteria (Figure 1). Time of shock (TS) was defined by the combined presence of mean arterial pressure (MAP) less than 60 mmHg for at least 15 mins and arterial lactate concentration ≥ 2.0 mmol/L. Pulmonary pressure was continuously monitored during lipopolysaccharide dose escalation to avoid severe pulmonary hypertension and right ventricle failure [SR3]. Lipopolysaccharide infusion was decreased to the immediately preceding dose in the case of mean pulmonary artery pressure (PAPm) ≥ 40 mmHg, increase of central venous pressure (CVP) ≥ 5 mmHg regarding to its baseline value, or when increase of CVP exceed pulmonary occlusion pressure (PAOP) by ≥ 3 mmHg. In the case of sustained PAPm ≥ 40 mmHg or persistently CVP exceeding PAOP by ≥ 3 mmHg for ≥ 1 hour, or when developing sustained hypotension, experimental model was stopped and discarded.

Shock (TS) was declared when mean arterial pressure (MAP) was steadily under 60 mmHg for at least 30 mins, and arterial and mesenteric lactate concentrations were ≥ 2.0 mmol/L. Lipopolysaccharide infusion was maintained up to 30 minutes after TS. Resuscitation was then started with fluids and norepinephrine as described elsewhere (37). Once MAP ≥ 75 mmHg was achieved, successive mini-fluid boluses of 4 mL·kg^{-1} of Lactate Ringer were

administered aiming to increase cardiac preload when positive fluid responsiveness was predicted (i.e., when pulse pressure [PPV] and stroke volume variations [SVV] were ≥ 15%), targeting arterial lactate levels < 2.0 mmol·L^{-1} and/or lactate decrease of at least 10% per 30 mins. All hemodynamics, respiratory parameters, and both systemic and regional blood gas analyses were performed at baseline, TS, when achieving MAP ≥ 75 mmHg, and then, every hour during the next six hours. Finally, euthanasia was performed at the end of the experiment according to the local regulations for animal research.

Two animals (one originally assigned to each experimental group) died before starting resuscitation: the first one developed profound shock secondary to severe right ventricular dysfunction while the second one developed sudden ventricular fibrillation. Both models were accordingly replaced following the original extended allocation sequence (which assumed up to 30% of potential model losses before to complete the protocol).

Calculating the Factors Influencing the PCO2:CCO2 Relationship

The following formulas for the two main factors (F_{bic} and F_{Hb}) and the three subfactors of F_{Hb} that influence the PCO_2/CCO_2 relationship were derived from Douglas' equation shown in Methods:

F_{bic}= 1 + $10^{(pH - pK')}$

F_{Hb}= 1 – 0.0289 × (Hb)/(3.352 – 0.456 × SO_2) × (8.142 – pH)

F_{Hb-Hb}= 0.0289 × Hb

F_{Hb-SO2}= 1/(3.352 – 0.456 × SO_2)

F_{Hb-pH}= 1/(8.142 – pH)

Microcirculatory measurements

We used a Side-stream dark-field (SDF) imaging device (Micro Scan; MicroVision Medical, Amsterdam, the Netherlands) to explore microcirculation. This portable video-microscope device uses a stroboscopic green light (around 530 nm wavelength), which is delivered to the tissues by multiple light-emitting diodes (LEDs). This wavelength of light is absorbed by hemoglobin of red blood cells, allowing their observation as dark cells flowing in the microcirculatory net while the light reflected by superficial layers does not reach the optics. As result of peripheral location of LEDs and the synchronization between light emission and camera frame rate, SDF provides a detailed visualization of open capillaries using a 5x objective and providing an on-screen magnification of x380.

Microcirculation at jejunal mucosa was evaluated by direct application of the SDF device through the surgical-prepared jejunostomy at five different points in an intestinal segment of at least fifteen centimeters, after careful removal of intestinal secretions by warm water and gentle aspiration. Light intensity and focus were manually adjusted until obtain the best quality in each case. Operator of SDF device was a well-trained researcher (G.O.T.) with expertise in acquiring images by SDF technique. At each time of measurements, we collected five sequences of video of 10 - 15 seconds each from different adjacent mucosa or serosa areas using a video card (MicroVideo; Pinnacle system, Mountain Views, CA, U.S.A.). These sequences of video were stored under a random number and later analyzed by two investigators blinded to the origin of sequences (G.A.G.G. and N.O.). For the analysis, the number of villi in each image were counted and individual villi microcirculation was semi-quantitatively classified according to its predominant blood flow, as either: normal-perfused (continuous blood flow), hypo perfused (intermittent or sluggish blood flow) or non-perfused (stopped blood flow). We quantified the percentage of normal-perfused villi (villi-PPV) in each video-sequence at each time-point of measurement [SR1].

The intra- and inter-observer variability for these methods have been studied in the past [SR2]. For the current study, the intra and inter-observer variability were determined by two observers, analyzing five sequences per each twenty acquired (N.O. and G.A.G.G.). Coefficient

of variability of the determination of one video sequence ranged from 3.8 to 6.0% (intra-observer) and from 3.5 to 6.5% (inter-observer) for the proportion of perfused vessels.

Supplementary Figures

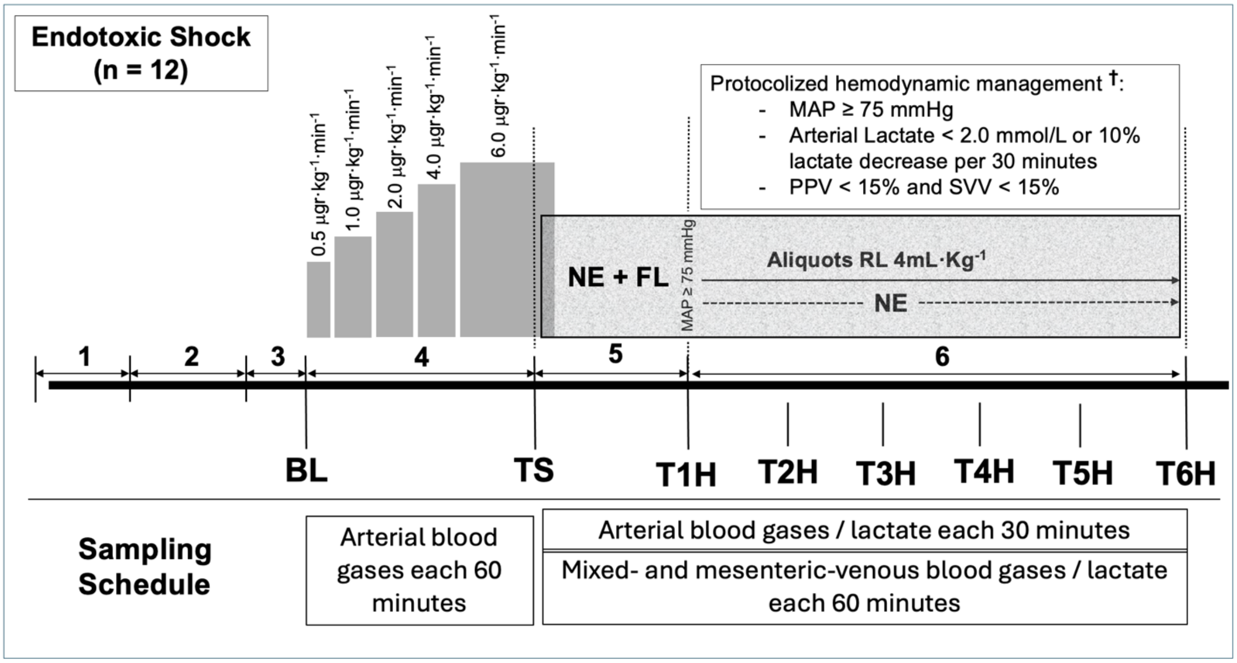

Figure S1. Experimental timeline.

1. Presurgical period (adaptation period, initial sedation, muscular paralysis, endotracheal intubation); 2. Surgical period (catheters insertion: carotid and pulmonary arteries, internal jugular veins, femoral artery—transpulmonary thermodilution system; laparotomy; insertion of ultrasound Doppler flow probes at the supra-celiac abdominal aorta and superior mesenteric artery; catheterization of superior mesenteric—spleen venous confluence; splenectomy; insertion of laser Doppler probes on jejunal mucosa and serosa surfaces; cystostomy; jejunal ostomy preparation for video-microscopy); 3. Stabilization period—at least 60 min; 4. lipopolysaccharide infusion—escalating dose up to 30 min after time of shock (TS); 5. Start of resuscitation (first hour): norepinephrine + fluid resuscitation up to achieve MAP ≥75 mmHg; 6. Measurements from 1 until 6 hr after shock (T1H, T2H, T3H, T4H, T5H, and T6H) resuscitation period—aliquots of 4 mL/kg of fluids according to protocolized hemodynamic management.

†Protocolized hemodynamic management: (a) MAP greater than or equal to 75 mm Hg; (b) Arterial lactate , 2.0 mmol/L or 10% lactate decrease per 30 min; (c) Pulse pressure variation - PPV - < 15% and stroke volume variation – SVV - < 15%

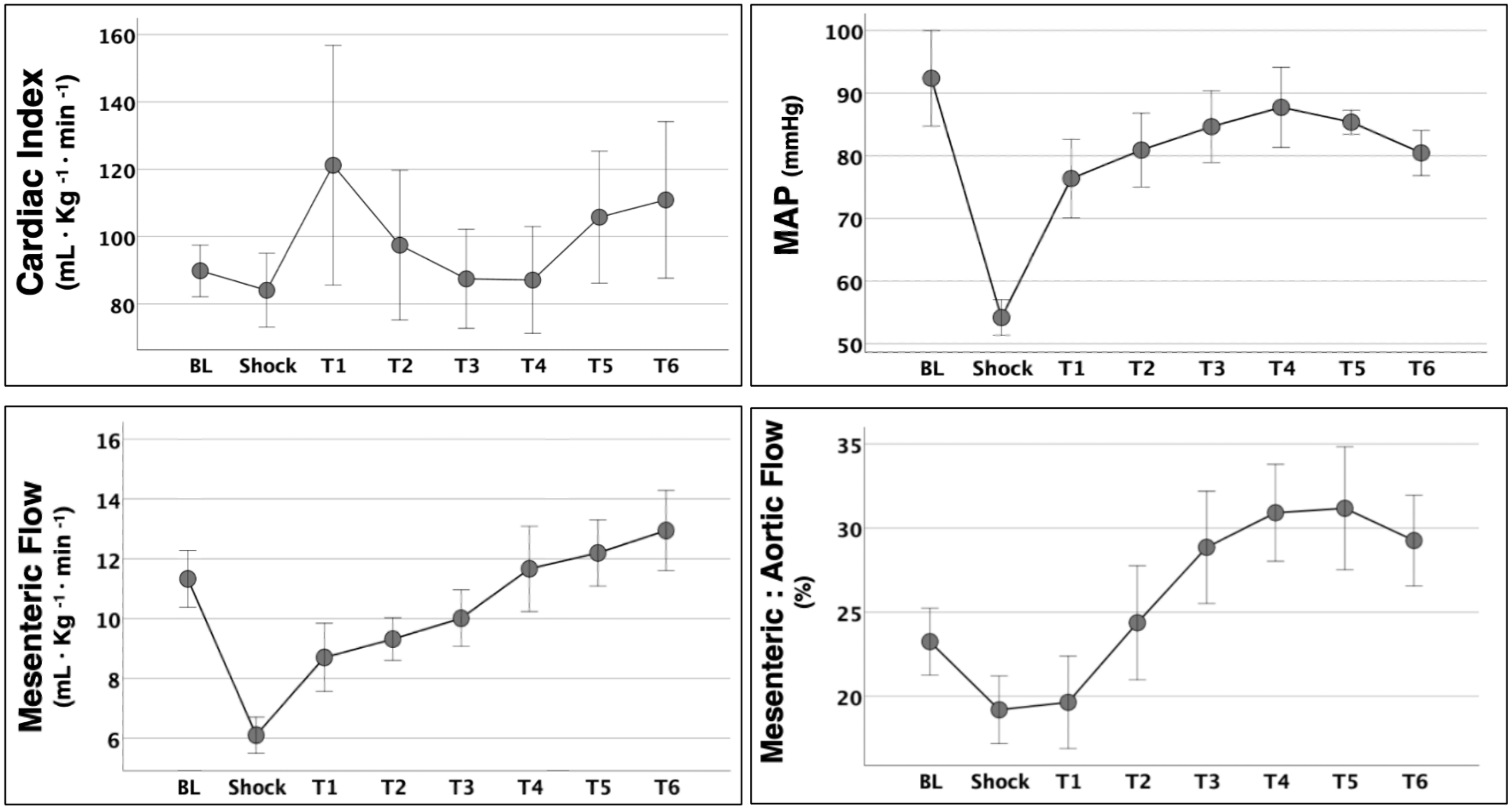

Figure S2. Time-course of systemic and splanchnic hemodynamics

Cardiac Index, in $mL \cdot kg^{-1} \cdot min^{-1}$; **MAP**, mean arterial pressure, in mmHg; **Mesenteric Flow**, flow at superior mesenteric artery in $mL \cdot kg^{-1} \cdot min^{-1}$; **Mesenteric: Aortic Flow**, the ratio between flow measured at superior mesenteric artery and at supra-celiac portion of abdominal aorta, in %

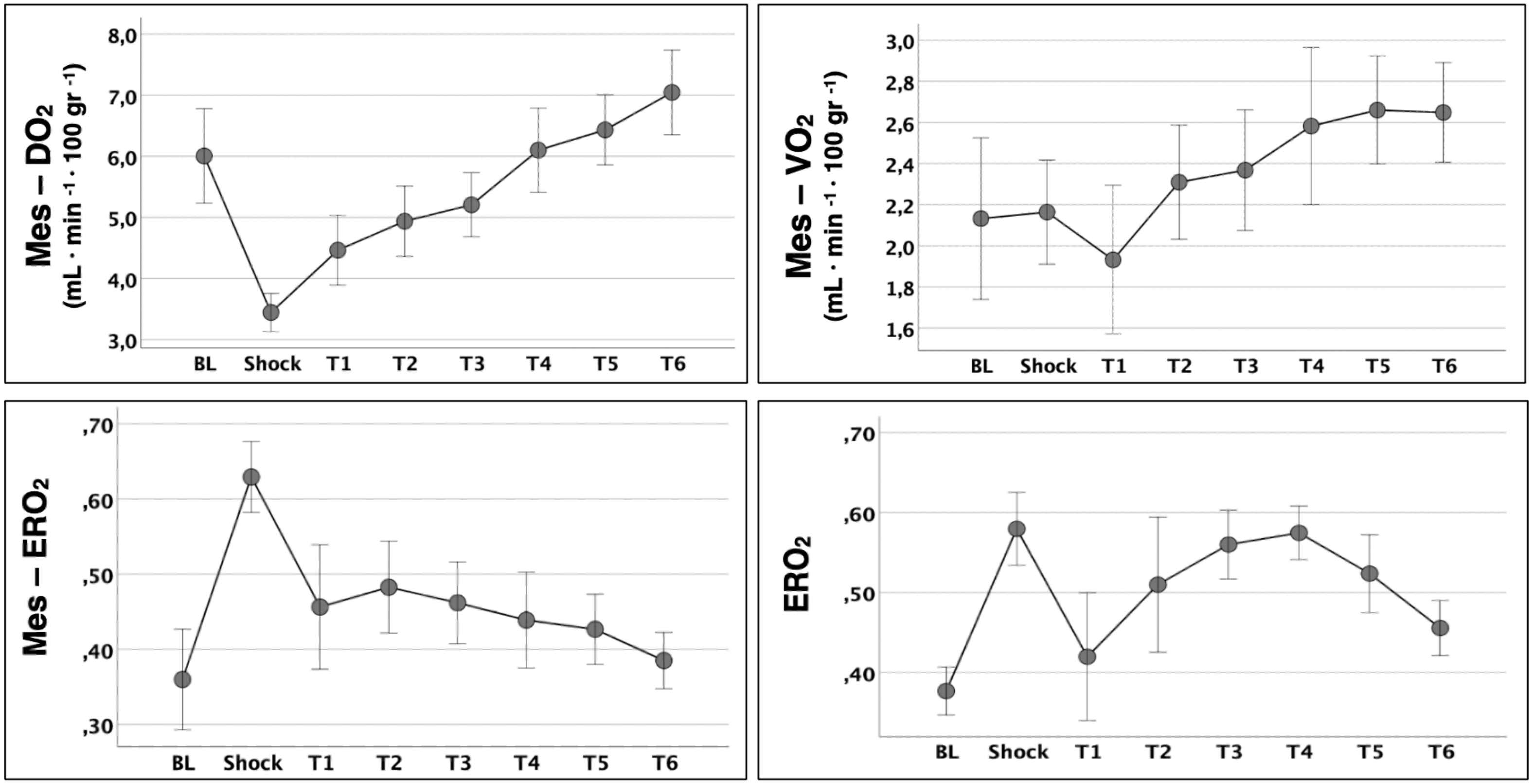

Figure S3. Time-course of splanchnic and systemic oxygen-derived parameters

Mes-DO$_2$, mesenteric oxygen delivery in mL·min^{-1}·100gr $^{-1}$; **Mes-VO$_2$**, mesenteric oxygen consumption in mL·min^{-1}·100gr $^{-1}$; **Mes-ERO$_2$**, mesenteric oxygen extraction ratio; **ERO$_2$**, systemic oxygen extraction ratio

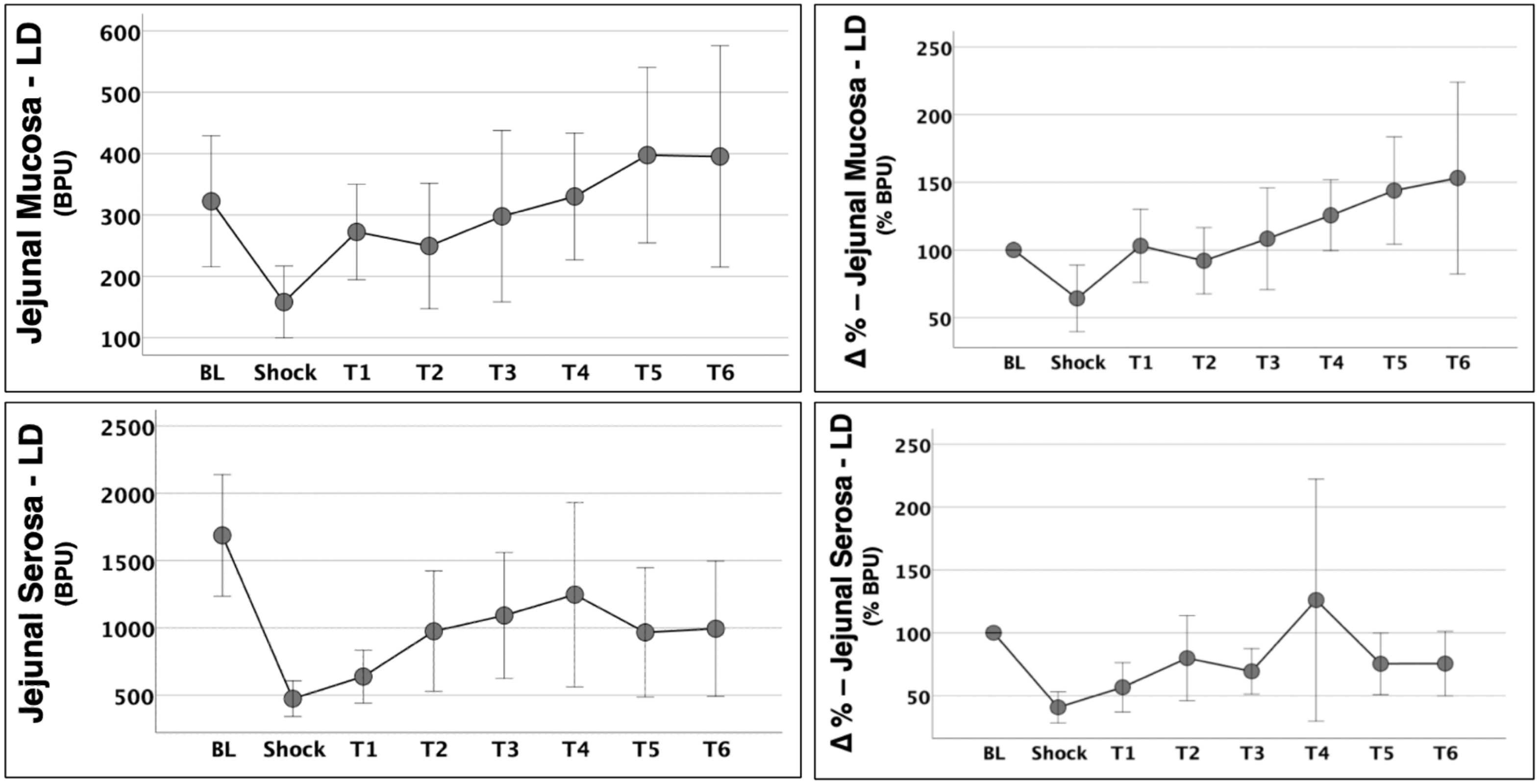

Figure S4. Time-course of jejunal microcirculatory blood flows

Jejunal Mucosa - LD, microvascular blood flow at jejunal mucosa in BPU (blood perfusion units); **Δ% – Jejunal Mucosa - LD**, % of variation of microvascular blood flow at jejunal mucosa compared with baseline, in % BPU (% of variation of blood perfusion units); **Jejunal Serosa - LD**, microvascular blood flow at jejunal serosa in BPU (blood perfusion units); **Δ% – Jejunal Serosa - LD**, % of variation of microvascular blood flow at jejunal serosa compared with baseline, in % BPU (% of variation of blood perfusion units)

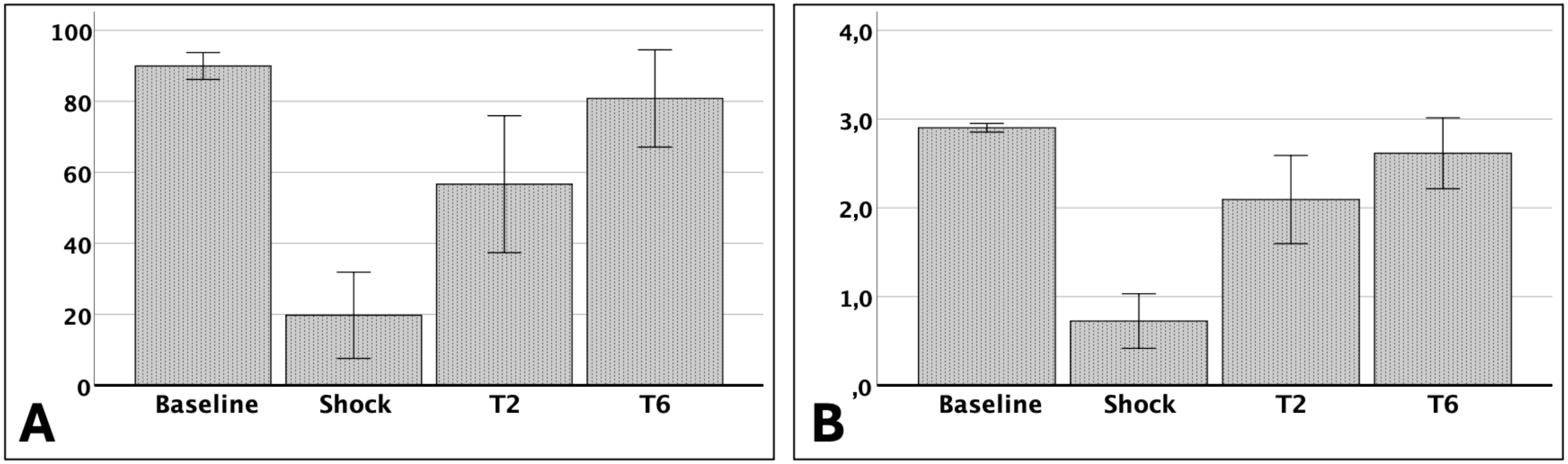

Figure S5. Time-course of jejunal microcirculatory blood flow assessed by SDF video-microscopy

Panel A. Percentage of jejunal villi with small-vessels continuous flow (% of vessels < 20 µm of diameter with continuous flow)

Panel B. Microvascular Flow Index

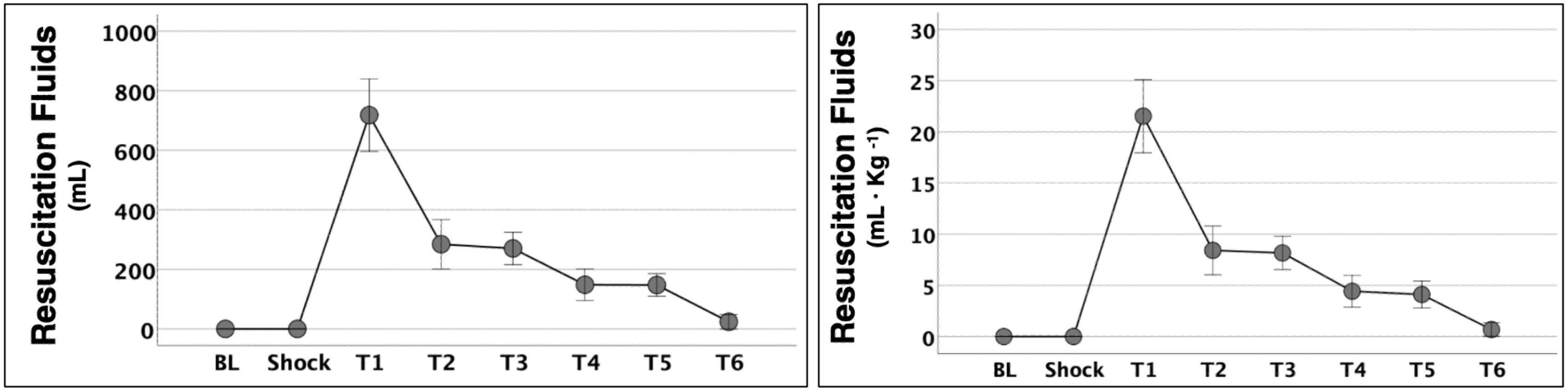

Figure S6. Time-course of resuscitation fluids

Resuscitation Fluids, in mL; **Resuscitation Fluids**, in in mL·kg^{-1}

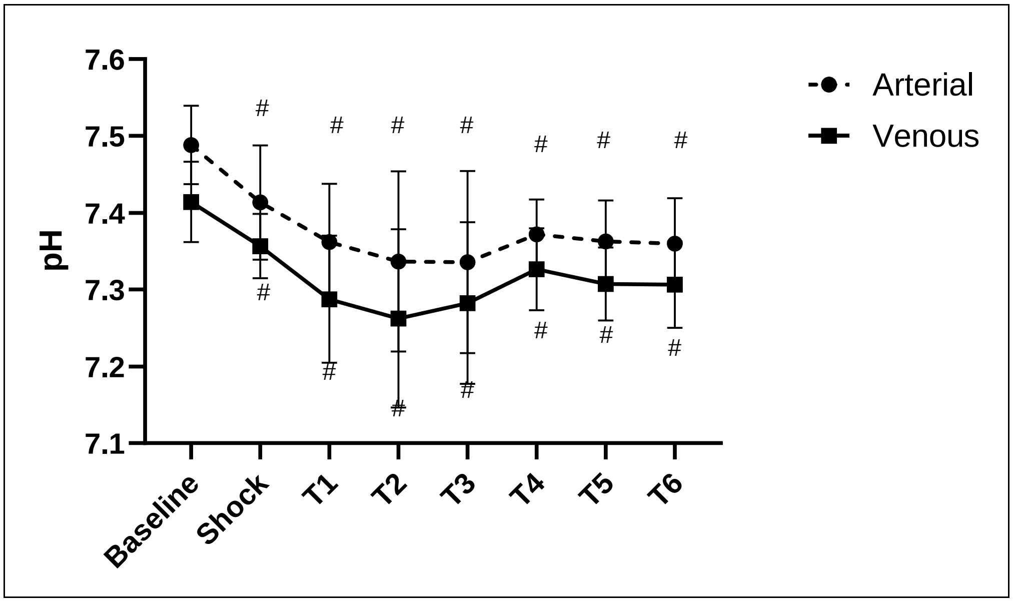

Figure S7. Time course of arterial and mesenteric venous pH.

#p < 0.0071 vs. baseline.

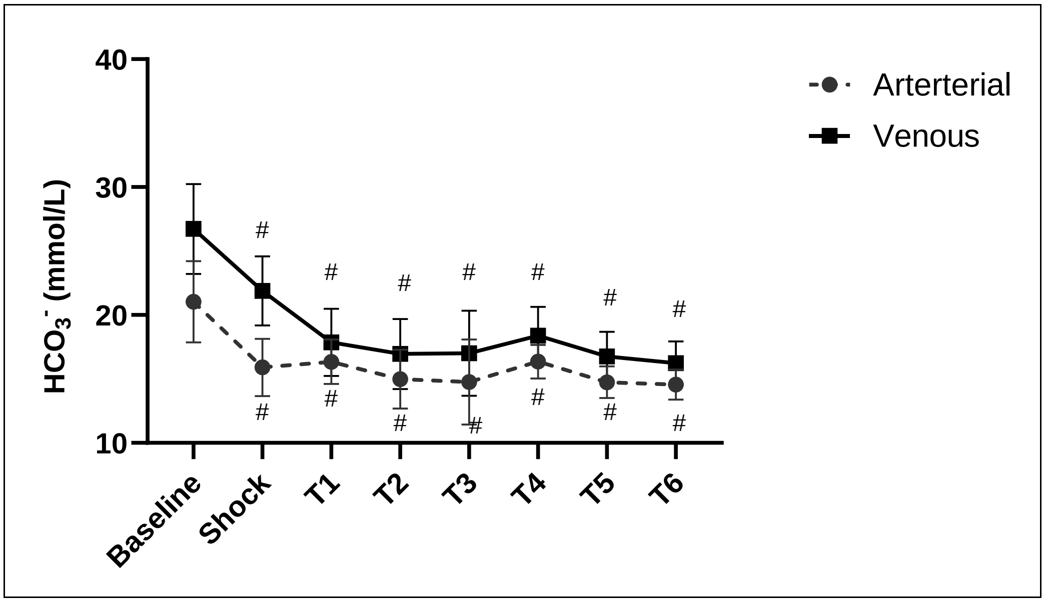

Figure S8. Time course of arterial and mesenteric venous bicarbonate (HCO_3^-).

#p < 0.0071 vs. baseline

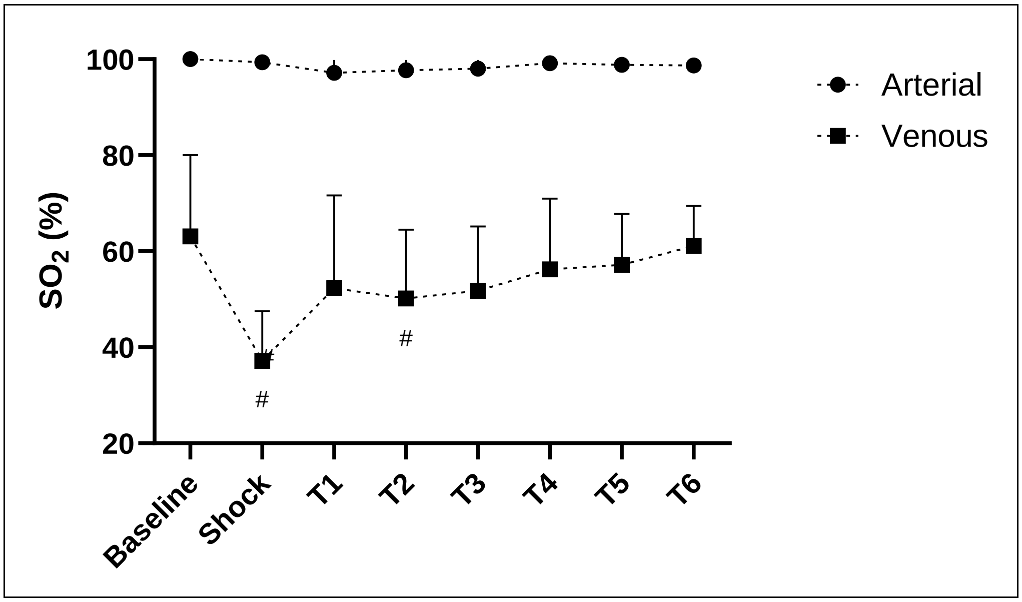

Figure S9. Time course of arterial and mesenteric venous oxygen saturation (SO_2).

#p < 0.0071 vs. baseline.

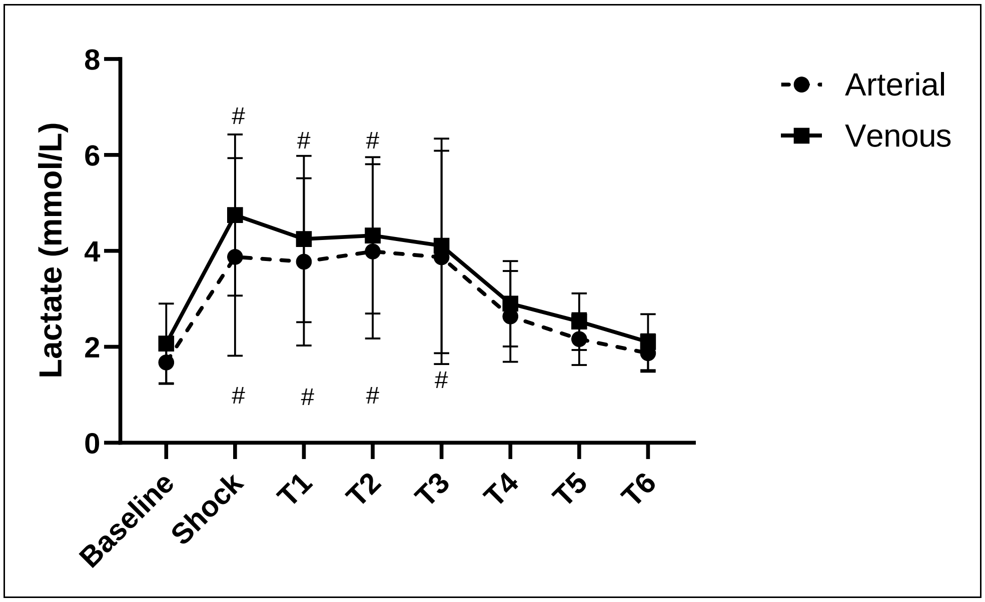

Figure S10. Time course of arterial and mesenteric venous lactate levels.

#p < 0.0071 vs. baseline.

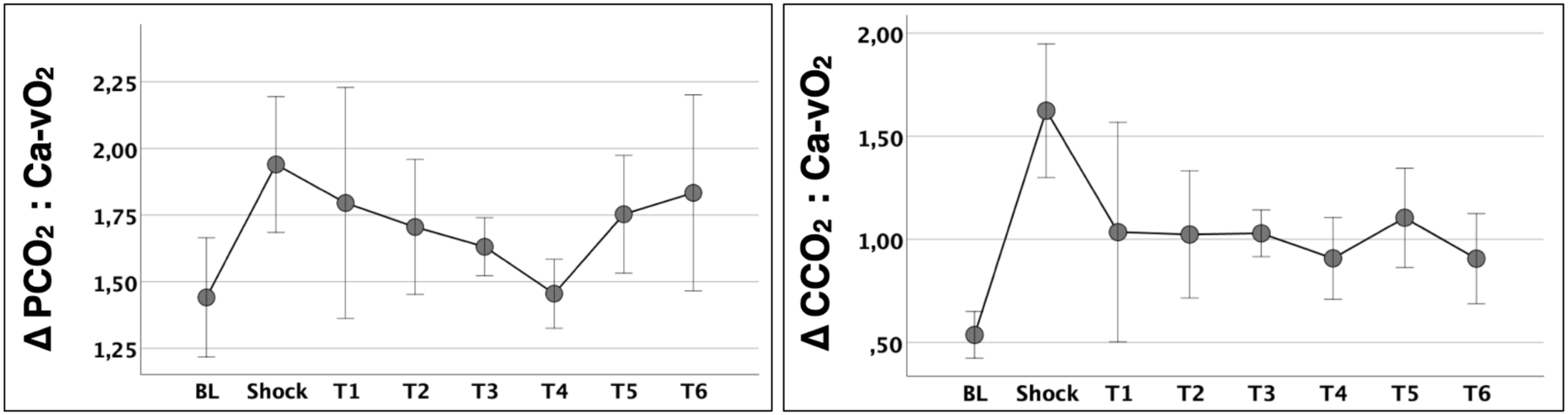

Figure S11. Time-course of $\Delta PCO_2 : \Delta PO_2$ and $\Delta PCO_2 : \Delta PO_2$ ratios.

$\Delta PCO_2 : \Delta PO_2$ denotes the mesenteric venous-to-arterial carbon dioxide pressure to arterial-venous oxygen difference ratio

$\Delta CCO_2 : \Delta PO_2$ denotes the mesenteric venous-to-arterial carbon dioxide content to arterial-venous oxygen difference ratio

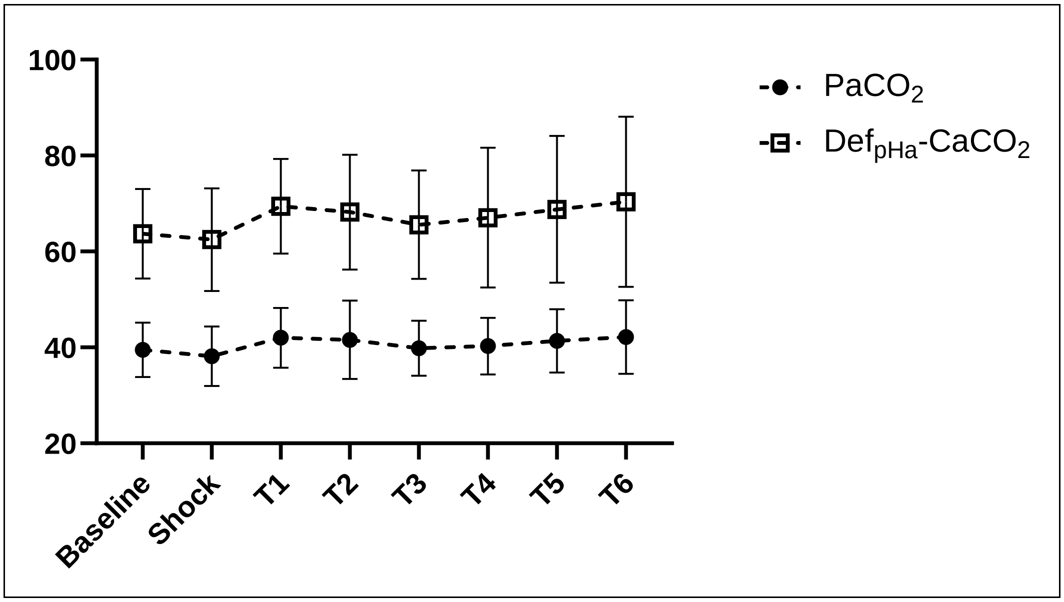

Figure S12. Time courses of arterial carbon dioxide pressure ($PaCO_2$) and default arterial pH arterial - carbon dioxide content (Def_{pHa}-$CaCO_2$).

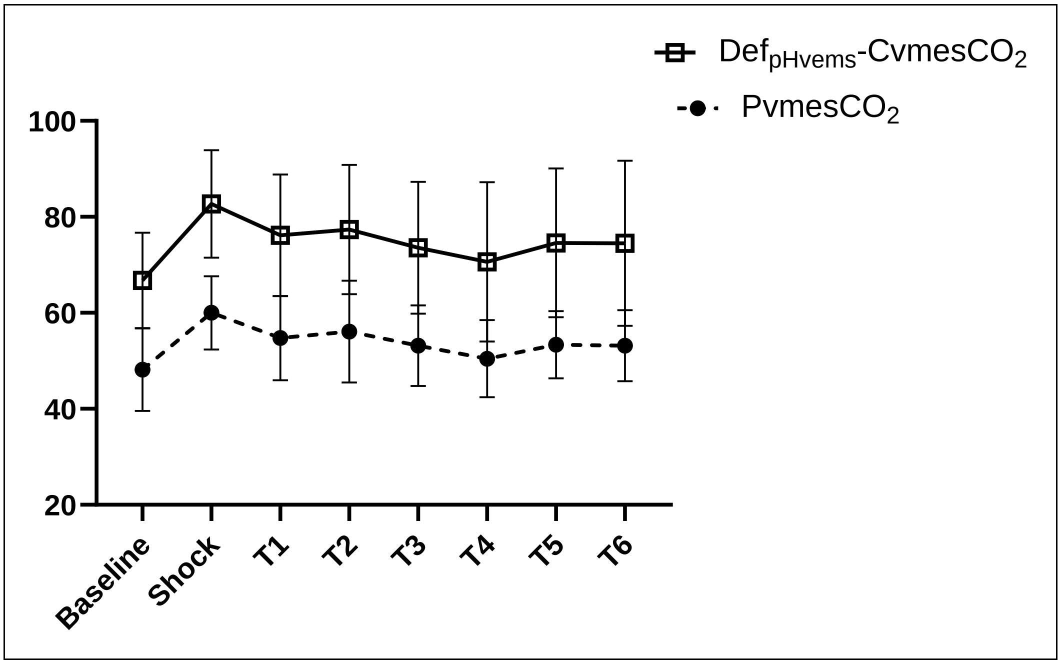

Figure S13. Time courses of mesenteric venous carbon dioxide pressure ($PvmesCO_2$) and default mesenteric venous pH mesenteric venous carbon dioxide content (Def_{pHvmes}-$CvmesCO_2$).

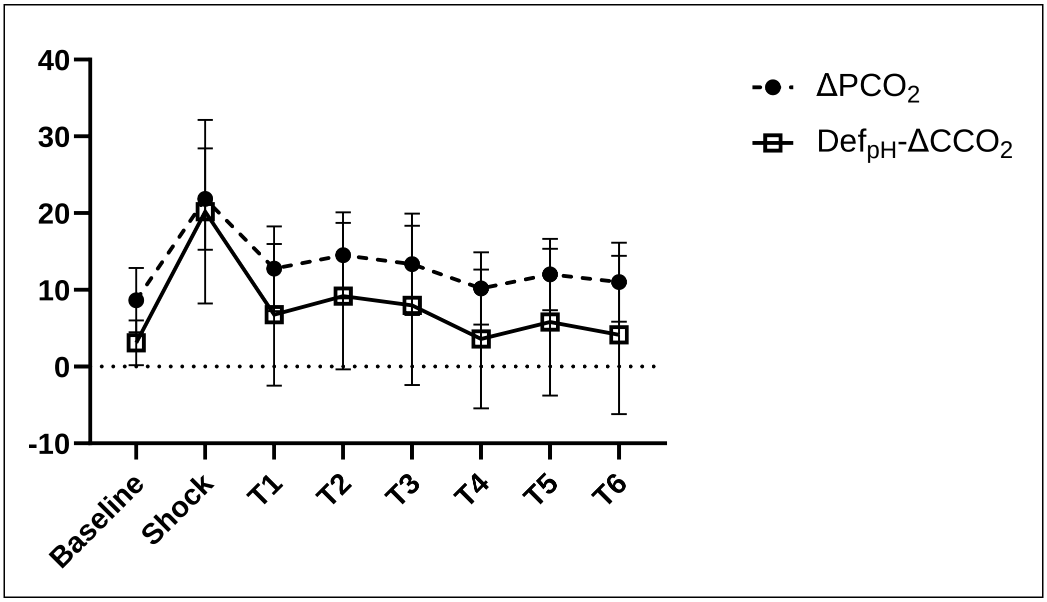

Figure S14. Time courses of the difference between mesenteric venous carbon dioxide pressure and arterial carbon dioxide pressure (ΔPCO_2) and default pH difference between mesenteric venous carbon dioxide content and arterial carbon dioxide content (Def_{pH}-ΔCCO_2).

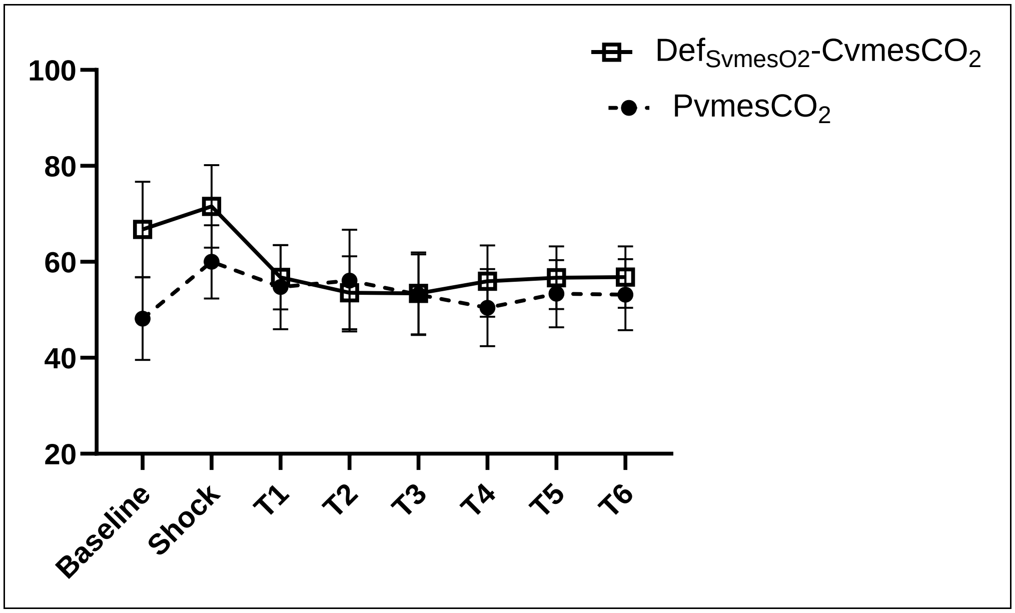

Figure S15. Time courses of mesenteric venous carbon dioxide pressure ($PvCO_2$) and default mesenteric venous oxygen saturation mesenteric venous carbon dioxide content ($Def_{SvmesO2}$-$CvmesCO_2$).

Supplementary Tables

Table S1. Hemodynamics and oxygen-derived parameters

	Baseline	Time-Shock	T1	T2	T3	T4	T5	T6	ANOVA	
									F	p
**General macro – Hemodynamics**										
**HR, beats.min^{-1}**	84 [72 – 99]	118 [97 – 149] *	133 [111 – 181] *	125 [110 – 146] *	117 [107 – 145] *	120 [103 – 123] *	133 [117 – 141] *	139 [120 – 170] *	7.2	<0.001
**SAP, mmHg**	109 [100 – 119]	69 [64 – 85] *	106 [92 – 118]	101 [93 – 109]	101 [94 – 108]	103 [93 – 110]	103 [97 – 109]	98 [91 – 107]	13.0	<0.001
**DAP, mmHg**	77 [71 – 90]	45 [41 – 51] *	65 [50 – 75]	65 [58 – 77]	71 [63 – 77]	68 [65 – 81]	71 [66 – 77]	66 [62 – 70]	8.7	<0.001
**MAP, mmHg**	92 [87 – 98]	57 [49 – 65] *	78 [67 – 90]	83 [72 – 96]	85 [78 – 95]	86 [76 – 92]	84 [81 – 87]	79 [75 – 84]	12.6	<0.001
**CVP, mmHg**	9 [7 – 10]	9 [6 – 11]	10 [6 – 11]	10 [8 – 10]	10 [8 – 11]	10 [7 – 11]	11 [7 – 12]	10 [7 – 12]	2.0	0.065

**PAOP, mmHg**	11 [8 – 12]	11 [9 – 12]	11 [8 – 12]	11 [9 – 12]	11 [10 – 13]	11 [10 – 12]	11 [10 – 12]	12 [9 – 13]	1.6	0.14
**PAPm, mmHg**	23 [18 – 28]	33 [31 – 40]	41 [32 – 51] *	42 [36 – 49] *	38 [31 – 45]	37 [31 – 38]	34 [32 – 37]	36 [33 – 41]	13.9	<0.001
**Cardiac Output, mL·Kg^{-1}·min^{-1}**	87.8 [80.9 – 96.7]	79.3 [67.2 – 98.9]	92.5 [67.8 – 108.9]	88.8 [73.0 – 100.6]	87.7 [64.7 – 100.9]	87.6 [60.9 – 122.6]	99.7 [73.9 – 130.0]	101.3 [71.5 – 138.7]	1.7	0.13
**PPV, %**		22.0 [17.0 – 24.0]	15.5 [13.0 – 24.0	16.0 [12.0 – 20.0]	16.0 [12.0 – 25.0]	13.5 [12.0 – 24.0]	15.0 [13.0 – 20.0]	16.0 [13.0 – 18.0]	1.8	0.10
**SVV, %**	18.0 [14.0 – 24.0]	16.0 [15.0 – 20.0]	17.0 [14.0 – 19.0]	15.0 [9.0 – 19.0]	14.0 [11.0 – 22.0]	15.0 [13.0 – 21.0]	16.0 [13.0 – 17.0]	13.0 [11.0 – 14.0]	0.8	0.63
**GEDVI, mL·m^2**	691 [573 – 735]	528 [453 – 638] *	508 [457 – 666] *	521 [435 – 600] *	514 [469 – 625]	489 [422 – 626] *	517 [422 – 626]	526 [400 – 606]	5.3	<0.001
**ELWI, mL·Kg^{-1}**	14.5 [14.0 – 16.4]	15.5 [14.0 – 16.4]	16.0 [14 – 17.0]	16.0 [14.0 – 17.0]	16.0 [14.0 – 18.0]	16.2 [14.0 – 18.0]	17.2 [16.0 – 19.0] *	16.6 [15.0 – 21.0] *	4.0	<0.001
**PVPI**	2.5 [2.4 – 2.8]	2.8 [2.5 – 3.2]	3.0 [2.7 – 3.7] *	3.0 [2.7 – 3.7] *	3.0 [2.8 – 3.7] *	3.0 [2.8 – 3.7] *	3.2 [2.6 – 4.1] *	3.2 [2.6 – 4.5] *	5.2	<0.001
**Regional and microvascular blood flows**										
**Abdominal Aortic Flow,**	49.6 [42.7 – 55.1]	34.5 [30.0 – 37.1] *	43.5 [41.4 – 52.0]	43.5 [37.9 – 53.9]	36.1 [32.3 – 49.7]	40.3 [31.4 – 55.6]	42.0 [32.1 – 54.1]	45.4 [39.7 – 58.8]	3.7	<0.001

**mL· min^{-1} · Kg^{-1}**										
**Mesenteric Flow,** **mL· min^{-1} · Kg^{-1}**	10.4 [8.8 – 11.7]	6.6 [5.7 –7.6] *	9.0 [7.3 – 10.1] *	9.2 [8.2 – 10.4]	9.4 [8.5 – 11.0]	10.7 [9.5 – 12.0]	11.3 [10.0 – 12.9]	12.2 [9.6 – 15.3]	15.8	<0.001
**Δ % Aortic Flow, %**	100.0 [100.0 – 100.0]	61.3 [58.9 – 100.0] *	93.3 [75.1 – 115.8]	85.2 [74.8 – 117.6]	79.3 [61.8 – 100.6]	77.0 [68.1 – 119.8]	84.3 [66.1 – 121.5]	87.9 [78.9 – 125.7]	3.8	<0.001
**Δ % Mesenteric Flow, %**	100.0 [100.0 – 100.0]	56.8 [50.0 – 78.1] *	90.9 [56.8 – 104.3]	84.5 [79.4 – 100.7]	94.6 [74.3 – 107.7]	104.6 [82.5 – 133.2]	116.1 [88.8 – 121.5]	119.1 [96.0 – 133.9]	18.2	<0.001
**Mesenteric to Aortic Flow ratio, %**	21.5 [19.2 – 26.0]	19.5 [16.4 – 22.2] *	17.1 [12.5 – 22.6]	20.3 [14.8 – 28.3]	24.9 [20.7 – 29.9]	27.9 [24.3 – 32.6] *	28.2 [19.2 – 34.2] *	28.3 [18.1 – 31.8] *	8.3	<0.001
**Laser Doppler Mucosa, BPU**	241 [189 – 320]	152 [104 – 234]	229 [160 – 289]	208 [186 – 260]	274 [146 – 302]	273 [210 – 359]	293 [222 – 406]	235 [164 – 395]	1.8	0.10
**Laser Doppler Serosa, BPU**	1122 [747 – 2260]	518 [238 –747] *	660 [331 – 885]	821 [292 – 1159]	990 [435 – 1624]	695 [243 – 1340]	774 [264 – 1095]	681 [265 – 1341]	2.8	0.01

**Δ % Laser Doppler Mucosa**	100.0 [100.0 – 100.0]	81.0 [17.2 – 101.3] *	102.6 [88.2 – 115.4]	103.6 [59.9 – 122.7]	101.9 [69.6 – 129.1]	127.6 [98.9 – 166.7]	130.6 [84.1 – 199.2]	90.3 [52.8 – 197.5]	2.1	0.05
**Δ % Laser Doppler Serosa**	100.0 [100.0 – 100.0]	35.0 [23.4 – 100.0] *	63.5 [28.4 – 87.1]	78.1 [30.8 – 141.1]	78.7 [30.8 – 141.1]	87.2 [26.5 – 128.9]	93.4 [29.1 – 127.4]	99.6 [29.3 – 121.4]	1.2	0.03
**% Villi-perfused**	93.8 [83.3 – 97.9]	17.2 [0.0 – 65.0] *		90.5 [32.7 – 99.0] *				97.9 [86.4 – 100.0]	20.4	<0.001
**MFI**	2.9 [2.8 – 3.0]	1.2 [0.0 – 1.5] *		2.9 [1.5 – 3.0]				3.0 [2.9 – 3.0]	26.0	<0.001
**Systemic and Regional Oxygen-derived variables**										
**Systemic DO_2, $mL \cdot Kg^{-1} \cdot min^{-1}$**	14.7 [12.5 – 15.9]	13.8 [11.0 – 19.3]	15.5 [10.7 – 19.0]	13.2 [10.6 – 17.3]	12.8 [10.4 – 14.5]	12.0 [10.5 – 17.0]	13.6 [12.1 – 19.5]	15.7 [10.6 – 19.6]	1.3	0.28
**Systemic VO_2,**	5.5 [4.7 – 6.9]	7.1 [5.3 – 10.0] *	6.4 [4.8 – 7.5]	5.9 [5.1 – 7.5]	5.9 [5.2 – 8.4]	6.2 [5.7 – 8.1]	7.3 [6.0 – 8.2]	6.1 [5.0 – 9.0]	2.7	0.02

**mL·Kg^{-1}·min^{-1}**										
**Systemic ERO$_2$**	0.41 [0.34 – 0.43]	0.54 [0.43 – 0.63] *	0.43 [0.32 – 0.47]	0.45 [0.37 – 0.64]	0.53 [0.41 – 0.59]	0.53 [0.47 – 0.63]	0.49 [0.39 – 0.56]	0.43 [0.36 – 0.49]	6.1	<0.001
**Splanchnic DO$_2$, mL·min^{-1}·100gr^{-1}**	5.5 [4.4 – 6.8]	3.5 [3.2 – 4.2] *	4.4 [4.0 – 5.3]	4.6 [4.3 – 5.0]	4.9 [3.9 – 5.7]	5.6 [4.3 – 7.1]	6.0 [5.2 – 7.0]	6.5 [5.5 – 7.2]	17.4	<0.001
**Splanchnic VO$_2$, mL·min^{-1}·100gr^{-1}**	2.3 [1.8 – 2.5]	1.9 [1.5 – 2.5] *	2.0 [1.4 – 2.6]	2.2 [1.6 – 2.7]	2.2 [1.7 – 2.8]	2.3 [1.8 – 3.1]	2.4 [2.2 – 2.8]	2.5 [2.3 – 2.6]	2.7	0.02
**Splanchnic ERO$_2$**	0.31 [0.26 – 0.37]	0.58 [0.44 – 0.66] *	0.51 [0.33 – 0.62] *	0.46 [0.33 – 0.54]	0.45 [0.39 – 0.55]	0.47 [0.39 – 0.53]	0.42 [0.40 – 0.49]	0.38 [0.33 – 0.42]	6.0	<0.001
**Resuscitation Fluids / Norepinephrine dose**										
**Norepinephrine, μgr·Kg·min^{-1}**	0.00 [0.00 – 0.00]	0.00 [0.00 – 0.00]	0.40 [0.22 – 0.65]	0.25 [0.10 – 0.35]	0.10 [0.05 – 0.20]	0.10 [0.03 – 0.30]	0.10 [0.03 – 0.23]	0.20 [0.07 – 0.38]	6.3	<0.001
**Resuscitation Fluids, mL**	0 [0 – 0]	0 [0 – 0] *	728 [442 – 975]	280 [184 – 398]	254 [150 – 354]	138 [60 – 207]	145 [130 – 195]	0 [0 – 0]	35.4	<0.001

**Resuscitation Fluids, mL·Kg^{-1}**	0.0 [0.0 – 0.0]	0.0 [0.0 – 0.0] *	17.1 [13.3 – 29.9]	8.0 [6.1 – 10.2]	8.0 [4.2 – 9.0]	4.2 [4.0 – 6.4]	4.0 [3.9 – 6.4]	0.0 [0.0 – 0.0]	36.5	<0.001

All data are presented as median [percentiles 25_75]. **HR**: Heart rate; **CO**: cardiac output; **MAP**: mean arterial pressure; **CVP**: central venous pressure; **PAOP**: pulmonary artery occlusion pressure; **PAPm**: mean pulmonary artery pressure; **PPV:** pulse pressure variation; **SVV:** stroke volume variation; **GEDVI:** global end-diastolic volume index; **ELWI:** extravascular lung water; **PVPI:** pulmonary vascular permeability index; **Δ % Aortic Flow:** % of aortic flow variation from baseline; **Δ % Mesenteric Flow: :** % of variation of the superior mesenteric artery flow from baseline; **BPU:** blood perfusion units; **Δ % Laser Doppler Mucosa**: % variation of jejunal mucosa microcirculatory blood flow assessed by laser doppler; **Δ % Laser Doppler Serosa:** % variation of jejunal serosa microcirculatory blood flow assessed by laser doppler; **% Villi-perfused:** % of jejunal villi with continuous flow assessed by video-microscopy; **MFI:** microcirculatory blood flow index; **Systemic DO$_2$:** systemic oxygen delivery; **Systemic VO$_2$:** systemic oxygen consumption; **Systemic ERO$_2$**: systemic oxygen extraction ratio; **Splanchnic DO$_2$:** splanchnic oxygen delivery; **Splanchnic VO$_2$:** splanchnic oxygen consumption; **Splanchnic ERO$_2$**: splanchnic oxygen extraction ratio.

***** S.N.K. test, $p < 0.05$ vs. baseline

Table S2. Comparisons between percentual disagreement by not accounting for mesenteric-venous pH and oxygen saturation variations

	**Disagreement – Def_{pHvmes} – $CvmesCO_2$ (%)**	**Disagreement – $Def_{SvmesO2}$ – $CvmesCO_2$ (%)**
**Baseline**	0	0
**Shock**	15.3 ± 10.8*	-0.39 ± 0.27
**T1**	39.8 ± 22.1*	-0.16 ± 0.26
**T2**	47.6 ± 41.9*	-0.19 ± 0.20
**T3**	40.8 ± 39.6*	-0.16 ± 0.25
**T4**	25.3 ± 20.2*	-0.13 ± 0.29
**T5**	31.1 ± 20.8*	-0.11 ± 0.26
**T6**	24.8 ± 21.3*	-0.12 ± 0.20

% Discrepancy = (default – actual) / actual value × 100.

*$p < 0.0071$ between %Discrepancy in Def_{pHvmes}-$CvCO_2$ and %Discrepancy in $Def_{SvmesO2}$-$CvCO_2$ (%)

Table S3. Factors describing the magnitude by which Hb, SO_2, or pH variations affect the PCO_2:CCO_2 relationship

	Arterial F_{Bic}	F_{Hb-Hb}	$F_{Hb-SaO2}$	Arterial F_{Hb-pH}	Mesenteric-venous F_{Bic}	$F_{Hb-SvmesO2}$	Mesenteric-venous F_{Hb-pH}
**Baseline**	1	1	1	1	1	1	1
**Shock**	0.86 ± 0.14#	1.12 ± 0.09#	1.00 ± 0.00	0.90 ± 0.09#	0.89 ± 0.07#	0.96 ± 0.02#	0.93 ± 0.05#
**T1**	0.76 ± 0.12#	1.02 ± 0.10	1.00 ± 0.00	0.84 ± 0.08#	0.76 ± 0.12#	0.98 ± 0.02#	0.86 ± 0.07#
**T2**	0.73 ± 0.15#	1.06± 0.13	1.00 ± 0.00	0.82 ± 0.09#	0.73 ± 0.13#	0.98 ± 0.02#	0.83 ± 0.08#
**T3**	0.73 ± 0.15#	1.06 ± 0.15	1.00 ± 0.00	0.82 ± 0.09#	0.77 ± 0.13#	0.98 ± 0.03	0.85 ± 0.08#
**T4**	0.78 ± 0.12#	1.04 ± 0.13	1.00 ± 0.00	0.85 ± 0.09#	0.84 ± 0.12#	0.99 ± 0.03	0.89 ± 0.08#
**T5**	0.77 ± 0.12#	1.01 ± 0.13	1.00 ± 0.00	0.84 ± 0.08#	0.81 ± 0.12#	0.99 ± 0.02	0.87 ± 0.08#
**T6**	0.77 ± 0.14#	1.01 ± 0.13	1.00 ± 0.00	0.84 ± 0.10#	0.81 ± 0.15#	0.99 ± 0.02	0.87 ± 0.09#

$\mathbf{F_{Bic}}$, pH factor of plasma and red cell bicarbonate; $\mathbf{F_{Hb\text{-}SO2}}$, SO_2 subfactor of hemoglobin (Hb) CO_2 binding; $\mathbf{F_{Hb\text{-}Hb}}$, Hb subfactor of Hb CO_2 binding; $\mathbf{F_{Hb\text{-}pH}}$, pH subfactor of Hb CO_2 binding. Factors describe the relative values of variables in the Douglas equation. In our animals, baseline factors values are F_{Bic} = 22.10 ± 2.59, $F_{Hb\text{-}Hb}$ 0.34 ± 0.04, $F_{Hb\text{-}SO2}$= 0.33 ± 0.00, and $F_{Hb\text{-}pH}$ = 1.38 ± 0.10 in mesenteric-venous blood and for arterial blood are F_{Bic}= 26.21 ± 3.04, $F_{Hb\text{-}Hb}$ = 0.34 ± 0.04, $F_{Hb\text{-}SO2}$= 0.34 ± 0.00, and $F_{Hb\text{-}pH}$ = 1.54 ± 0.13. Values show the relative changes in factors from their baseline values. The further the values differ from 1.00, the greater their influence on the PCO_2:CCO_2 relationship.

Supplementary References

SR1. Ospina-Tascón GA, García Marin AF, Echeverri GJ, Bermudez WF, Madriñán-Navia H, Valencia JD, Quiñones E, Rodríguez F, Marulanda A, Arango-Dávila CA, Bruhn A, Hernández G, De Backer D. Effects of dobutamine on intestinal microvascular blood flow heterogeneity and O_2 extraction during septic shock. J Appl Physiol (1985). 2017 Jun 1;122(6):1406-1417. doi: 10.1152/japplphysiol.00886.2016.

SR2. De Backer D, Creteur J, Preiser JC, Dubois MJ, Vincent JL.
Microvascular blood flow is altered in patients with sepsis. Am J Respir Crit Care Med. 2002 Jul 1;166(1):98-104. doi: 10.1164/rccm.200109-016oc.PMID: 12091178

SR3. T Uchida, K Ichikawa, K Yokoyama, C Mitaka, H Toyooka, K Amaha. Inhaled nitric oxide improved the outcome of severe right ventricular failure caused by lipopolysaccharide administration. Intensive Care Med. 1996 Nov;22(11):1203-6. doi: 10.1007/BF01709337.

NOTE: Please save this file locally before filling in the table, DO NOT work on the file within your internet browser as changes will not be saved. Adobe Acrobat Reader (available free here) is recommended for completion.

The ARRIVE guidelines 2.0: author checklist

The ARRIVE Essential 10

These items are the basic minimum to include in a manuscript. Without this information, readers and reviewers cannot assess the reliability of the findings.

Item		Recommendation	Section/line number, or reason for not reporting
**Study design**	1	For each experiment, provide brief details of study design including:	
		a. The groups being compared, including control groups. If no control group has been used, the rationale should be stated.	Page 7 & 8
		b. The experimental unit (e.g. a single animal, litter, or cage of animals).	Page 5
**Sample size**	2	a. Specify the exact number of experimental units allocated to each group, and the total number in each experiment. Also indicate the total number of animals used.	Page 5
		b. Explain how the sample size was decided. Provide details of any *a priori* sample size calculation, if done.	N/A
**Inclusion and exclusion criteria**	3	a. Describe any criteria used for including and excluding animals (or experimental units) during the experiment, and data points during the analysis. Specify if these criteria were established *a priori*. If no criteria were set, state this explicitly.	Page 8
		b. For each experimental group, report any animals, experimental units or data points not included in the analysis and explain why. If there were no exclusions, state so.	N/A
		c. For each analysis, report the exact value of *n* in each experimental group.	Page 12-16 and supplemental
**Randomisation**	4	a. State whether randomisation was used to allocate experimental units to control and treatment groups. If done, provide the method used to generate the randomisation sequence.	N/A
		b. Describe the strategy used to minimise potential confounders such as the order of treatments and measurements, or animal/cage location. If confounders were not controlled, state this explicitly.	N/A
**Blinding**	5	Describe who was aware of the group allocation at the different stages of the experiment (during the allocation, the conduct of the experiment, the outcome assessment, and the data analysis).	N/A
**Outcome measures**	6	a. Clearly define all outcome measures assessed (e.g. cell death, molecular markers, or behavioural changes).	Page 7-12
		b. For hypothesis-testing studies, specify the primary outcome measure, i.e. the outcome measure that was used to determine the sample size.	Page 5 & 12
**Statistical methods**	7	a. Provide details of the statistical methods used for each analysis, including software used.	Page 12 & 13
		b. Describe any methods used to assess whether the data met the assumptions of the statistical approach, and what was done if the assumptions were not met.	Page 12
**Experimental animals**	8	a. Provide species-appropriate details of the animals used, including species, strain and substrain, sex, age or developmental stage, and, if relevant, weight.	Page 5
		b. Provide further relevant information on the provenance of animals, health/immune status, genetic modification status, genotype, and any previous procedures.	N/A
**Experimental procedures**	9	For each experimental group, including controls, describe the procedures in enough detail to allow others to replicate them, including:	
		a. What was done, how it was done and what was used.	Page 7 & 8
		b. When and how often.	Page 7 & 8
		c. Where (including detail of any acclimatisation periods).	Page 7 & 8
		d. Why (provide rationale for procedures).	Page 11 & 12
**Results**	10	For each experiment conducted, including independent replications, report:	
		a. Summary/descriptive statistics for each experimental group, with a measure of variability where applicable (e.g. mean and SD, or median and range).	Page 13-16
		b. If applicable, the effect size with a confidence interval.	N/A
